# Assessment of potentially toxic element contents in chickens and poultry feeds from Bangladesh markets: Implications for human health risk

**DOI:** 10.1016/j.toxrep.2024.101706

**Published:** 2024-08-10

**Authors:** Shamim Al Mamun, Mohammad A. Islam, Shamshad B. Quraishi, Mohammad M. Hosen, Brett H. Robinson, Ismail M.M. Rahman

**Affiliations:** a Department of Environmental Science and Resource Management, Mawlana Bhashani Science and Technology University, Santosh, Tangail 1902, Bangladesh; b Analytical Chemistry Laboratory, Chemistry Division, Atomic Energy Centre, Bangladesh Atomic Energy Commission, Dhaka 1000, Bangladesh; c School of Physical and Chemical Sciences, University of Canterbury, 20 Kirkwood Ave, Ilam, Christchurch 8041, New Zealand; d Institute of Environmental Radioactivity, Fukushima University, 1 Kanayagawa, Fukushima City, Fukushima 960-1296, Japan

**Keywords:** Potentially toxic elements, Contamination, Poultry feed, Health hazards

## Abstract

Chicken (*Gallus domesticus*) is a significant source of animal protein for the people of Bangladesh. However, anthropogenic activity may contaminate chicken meat with potentially toxic elements (PTEs) despite the nutritional benefits. Current work aims to determine the accumulated content of PTEs (Pb, Cd, Cr, As, and Hg) in chickens and poultry feeds commercially sold in Bangladesh markets and compare with WHO, FAO, EU, EC, FSANZ standards. Three different chicken varieties, *native* (local variety, freehand raised), *poultry* (raised for meat only), and *layer* chicken (commercially raised for eggs and later used for meat), were investigated, and commercial poultry feeds were used to raise the latter two varieties. The Pb, Cd, Cr, As, and Hg contents (mg kg^–1^ fresh weight (f.w.) were 0.481–1.067, 0.025–0.118, 0.069–0.319, 0.007–0.071, 0.002–0.019, respectively. In addition, associated health risks due to the PTEs in different varieties of chicken organs, e.g., meat, liver, and kidney, were evaluated. The study suggests that the poultry feeds should be carefully monitored regarding PTEs content to avoid potential human health risks due to chicken consumption in Bangladesh.

## Highlights

• Lead and chromium concentrations exceeded EU guidelines in meat, liver and kidney of native, poultry and layer chicken samples.
• Cadmium concentrations in chicken livers and chicken kidneys of native and layer chickens exceeded the EU guidelines.
• 11-month-old chickens had higher cadmium in meat, liver, and kidneys than 1-month-olds, making younger ones safer to eat.
• Older free-range chickens' meat, liver, and kidneys may be unsafe if they consume yard vegetables high in heavy metals.

## Graphical Abstract

**Figure:**
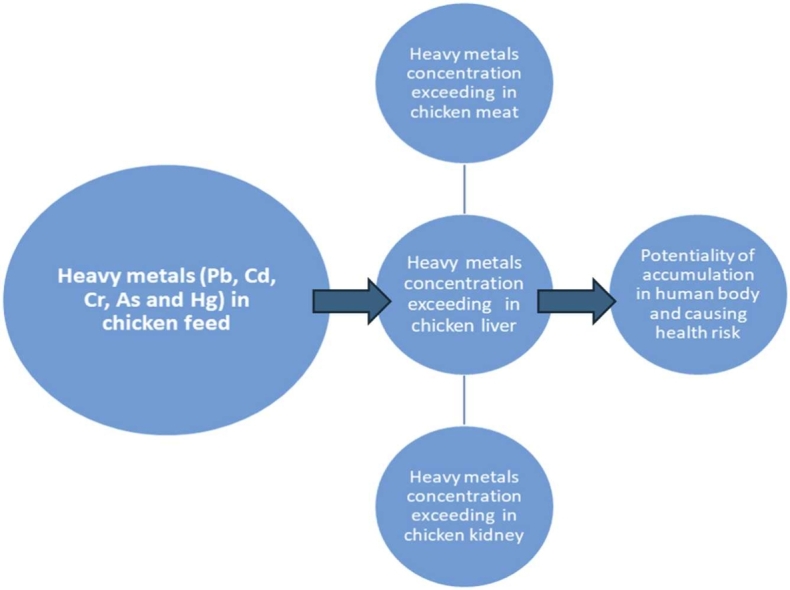

## 1 Introduction

Industrial sites in Bangladesh, including tanneries, ceramic factories, textile dyeing facilities, and sulfuric acid manufacturers, are known to release excessive amounts of heavy metals such as cadmium, lead, mercury, chromium, manganese, nickel, copper, and zinc. This has resulted in contamination of soil, vegetables, and water resources in the area [44]. Certain toxic elements, particularly lead, cadmium, chromium, arsenic, and mercury, can accumulate in food chains. These elements pose significant health risks due to their toxicity and ability to bioaccumulate and biomagnify in living organisms [17]. These potentially toxic elements can cause both immediate and long-term health issues. Their effects include disrupted metabolism in the kidneys and liver, as well as disorders affecting the cardiovascular, central nervous system (CNS), and skeletal systems [66], [52], [11].

Chickens (*Gallus domesticus*) are widely consumed in many countries, including Bangladesh, where annual consumption reaches 171,000 tons. Their popularity stems from low cost, easy availability, high protein content, and presence of micronutrients. Chickens are also extensively farmed in Bangladesh. [36]. Despite their nutritional benefits, chicken meat quality can be compromised by contamination with potentially toxic elements resulting from various human activities [18]. While some studies have examined element contamination in chicken feed and accumulation in chicken organs, as well as potential human health risks from consuming different chicken parts [39], significant concerns about trace element residues in chicken meat have been investigated in multiple countries and regions [76], [51], [72], [85]. Poultry production requires large-scale feed production. In Bangladesh, the annual feed demand is approximately 8.0 million metric tons, with poultry farms being the largest consumers. Corn comprises 50–60 % of poultry feed raw ingredients [77]. Various raw materials are used in poultry feed production (Islam et al., 2008). Commercial broiler chicken feed contains plant-based products, corn, wheat, soybean meal, small fish (often from the sea), chicken organ waste, animal fat, limestone, monocalcium phosphate, salts, and vitamin-mineral supplements [70]. Chickens are primarily exposed to potentially toxic elements through drinking water, litter, and contaminated feed [30]. Furthermore, some unethical businesses use toxic tannery waste in poultry feed production, significantly increasing contamination. These contaminated feeds can lead to the transfer of potentially toxic elements to chickens through the food chain, accumulating in their liver, kidney, muscle, and bones. Consequently, food products made from these chickens and contaminated cereals may contain potentially toxic elements [35]. Kidneys and liver are key organs for monitoring element contamination in animals due to their role in eliminating potentially toxic elements from the body [1]. Miranda et al. [59] noted that kidneys, like the liver, accumulate significant amounts of potentially toxic elements.

Lead, a highly toxic and non-essential element, is found in all mammalian tissues and organs [45]. High lead levels in poultry products primarily result from contaminated feed and water sources. Cadmium is absorbed through the gastrointestinal tract and lungs, transported via blood, and accumulates in various tissues and organs, particularly the kidneys and liver, due to its slow elimination rate from these organs [48]. Arsenic can be taken up by organisms that are either consumed by humans or used in livestock and poultry feed [16]. Some feed additives, such as roxarsone, increase arsenic content in chicken meat [82]. In Bangladesh, most poultry farms use shallow well water, which typically contains more arsenic than deep well water [26]. Arsenic accumulation in animal organs depends on their diet [42]. Mercury accumulation in broiler chicken tissues may result from feeds made from small sea fish and low-quality grains [70]. Mercury concentrations exceeding the maximum permissible limit (30 μg/kg) in food can cause serious health issues, including vision and hearing loss, and mental retardation [45].

Several studies have identified dangerous levels of chromium in poultry meat and eggs. A 2007 study by Dhaka University and the Bangladesh Council for Scientific and Industrial Research (BCSIR) found chromium levels in eggs and poultry meat exceeding acceptable limits. The highest chromium content in solid tannery waste was measured at 3.2 % [57]. Multiple studies indicate that tannery waste contains high levels of chromium, which could enter the food chain if used in poultry or fish feed. Consuming fish, chicken, or eggs contaminated in this way could lead to cancer or liver and kidney diseases.

In light of these concerns, an experiment was conducted to measure the concentrations of potentially toxic elements (lead, cadmium, chromium, arsenic, and mercury) in feed, meat, liver, and kidney samples from native, poultry, and layer chickens in Santosh, Tangail, Bangladesh. The study also aimed to assess the associated human health risks from exposure to these potentially toxic elements.

## 2 Materials and method

### 2.1 Sample introduction

#### 2.1.1 Chicken sample

Native chickens, also known as deshi (local) chickens (Nondescript Deshi (ND) [67]) are breeds of *Gallus domesticus* raised for both meat and eggs. These chickens are commonly kept by rural families in Bangladesh and typically forage for food around the household without being given commercial feed. Poultry chickens (Cobb 500) [19] also known as broiler on the other hand, are specifically bred for meat production. To ensure high-quality meat, these chickens are fed commercial feed and are usually white-feathered.

Layer (Orrpington breeds) [29] chickens (laying chicken) are those raised primarily for egg production. Like poultry chickens, they are also fed commercial feed. A brief overview of the weight and age of these different chicken types is provided in supplementary Table S1 (not included in this text). For the study about 100 g (f.w.) of meat (chest), whole liver, and whole kidney samples were obtained directly from the farmers who raised these chickens of the above mentioned breeds of native, layer and poultry. The samples were dried at 70˚C for seven days until a constant weight was observed. There were three replications for each sample.

#### 2.1.2 Chicken feed

The chicken feed samples were collected from the same area for analysis, which are normally given to the commercial chicken eg. poultry or layer chicken. Native chickens are generally reared on the nearby vegetable or crop fields, yard insects, crop residues etc. The three samples of chicken feed were randomly entitled as chicken feed 1(CF1), chicken feed 2 (CF2) and chicken feed 3 (CF3). To avoid the probable economic loss of the companies due to the publication of the manuscript, we have decided not to use the companies name. The marketable feeds of broiler chicken consist of mixtures of plant-related products, as well as other constituents such as soybean meal, wheat corn (particulary taken from the ocean), waste organs of chicken, animal fat, limestone, monocalcium hosphate, salts and vitamin-mineral ingredients [70]. Raw materials eg. rice bran, rice polish, solvent extracted rice, and wheat bran and molasses are generally used to make poultry feeds. Minerals such as calcium, phosphorus, trace minerals (such as Fe, Zn, Mn, Cu, CO and mg) and vitamins A, D, E, K, and B complex are also added in poultry feed [75].

### 2.2 Analytical methods

#### 2.2.1 Instruments and reagents

An atomic absorption spectrometer (AAS) (Model: AA240FS, Varian, Australia) with an auto sampler was used to detect potentially toxic elements (cadmium, lead, and chromium) in chicken samples. For arsenic and mercury detection, cold vapor AAS and hydride generation AAS methods were employed using a Varian AA240FS equipped with a hydride vapor generator. Hollow cathode lamps for lead, cadmium, chromium, arsenic, and mercury were used according to manufacturer guidelines.

Atomic signals were measured using peak area mode for cadmium, lead, and chromium, and integration mode for arsenic and mercury. Sample digestion occurred in a digestion chamber. Daily standard solutions were prepared by diluting 1000 μg/L^−^ stock solutions with 1 % (w/w) suprapur grade nitric acid (Merck, Germany).

For mercury and arsenic detection, hydrochloric acid (Merck, Germany), sodium borohydride, and sodium hydroxide were used as reductants. Potassium iodide and ascorbic acid (both from Merck, Germany) were used as additional reductants in arsenic sample preparation.

All glassware was cleaned by soaking in 20 % nitric acid overnight and rinsing three times with deionized water. Deionized water from an E-pure system (Thermo Scientific, USA) was used for all solutions, along with analytical-grade reagent chemicals.

#### 2.2.2 Sample preparation and digestion

Chicken samples were cut into small pieces, and approximately 30 g of each fresh sample was placed in petri dishes for oven drying. Samples were dried at 70°C in an electric hot air oven until reaching a constant weight. The dried samples were then ground into a homogeneous powder using a manual pestle and mortar, and stored in airtight plastic bags.

For analysis, 1.0 g of each dried sample was accurately weighed into a 50 ml beaker (Ref. to BSA 2243-CW, Germany). 10 ml of concentrated 69 % nitric acid (Merck, Germany) was added to each beaker in a digestion chamber, covered with a watch glass, and placed on a hotplate digestion system. The hotplate temperature was maintained between 60°C and 90°C for digestion. Samples were digested for 8 hours until the solution became colorless.

After cooling, the digested samples were filtered using Whatman no. 41 filter paper and diluted to 20 ml with deionized water. The samples were then refrigerated. For quality control and accuracy checks, a blank, two spike samples, and one standard reference material were digested similarly. A recovery study was conducted by spiking meat, liver, kidney, and feed samples with 4 mg/kg lead, 2 mg/kg cadmium, 2 mg/kg chromium, and 500 ppb arsenic. Triplicate experiments of spiked samples and blanks were performed through the digestion reaction. The digested and processed samples were analyzed for potentially toxic elements using an atomic absorption spectrophotometer (Varian, Australia; Model: AA240FS) at the Analytical Chemistry Laboratory, Chemistry Division, Atomic Energy Centre, Bangladesh Atomic Energy Commission, Dhaka, Bangladesh.

#### 2.2.3 Quality control and accuracy of the method

Quality control procedures were rigorously followed to validate all results. Each batch of sample preparation included reagent blanks to eliminate environmental effects, repeated samples to ensure precision, and spiked samples to verify method recovery. Results were only accepted when spike recovery fell within ±20 % of the expected values. The method's accuracy was confirmed using DORM-2 (Dogfish muscle), a certified reference material (CRM) from the National Research Council of Canada (NRCC). Specifically, the CRM was tested for chromium content. The certified value was 34.7 ± 5.55 mg/kg, while the obtained result was 32.04 ± 0.81 mg/kg, yielding a 92.3 % recovery rate.

### 2.3 Data calculations

#### 2.3.1 Estimates daily intakes (EDIs) of potentially toxic elements

The estimated daily intakes (EDIs) of potentially toxic elements (lead, cadmium, chromium, arsenic, and mercury) from consuming native, poultry, and layer chicken organs (meat, liver, and kidney) are based on the concentration of these elements in the respective organs, calculated on a fresh weight basis.

To determine the daily intake of potentially toxic elements by consumers from chicken samples, the following equation was used:

$$\mathrm{EDI}=\frac{\mathrm{CM}\times \mathrm{IR}}{\mathrm{WAB}}$$

Where: IR = ingestion rate of chicken organs (49.5 g/person/day) for the people of Bangladesh, as reported in the "Report of the household income and expenditure survey 2010" [13] WAB = average body weight (70 kg) CM = mean concentration (mg/kg, fresh weight) of potentially toxic elements in chicken organs (meat, liver, and kidney).

#### 2.3.2 Target hazard quotient (THQ)

The target hazard quotient (THQ) is used to assess the potential non-carcinogenic health risk level caused by pollutant exposure. In this study, THQ was calculated using the United States Environmental Protection Agency (USEPA) Region III Risk-Based Concentration Table to evaluate human health risks from consuming various chicken organs [80]. The following equation was used to estimate the target hazard quotient (THQ):

$$\mathrm{THQ}=\frac{\mathrm{EF}\times \mathrm{ED}\times \mathrm{IR}\times \mathrm{CM}}{\mathrm{WAB}\times \;AT_{n}\times \mathrm{RfD}}\times \;10^{-3}$$

Where:

THQ = target hazard quotient
ED = exposure duration (30 years for non-cancer risk, as recommended by USEPA, 2018)
EF = exposure frequency (365 days/year)
WAB = average body weight (70 kg) [80], [13]
CM = concentration of potentially toxic elements in chicken organs (mg/kg, fresh weight)
IR = ingestion rate of various chicken organs (49.5 g/person/day) [13]
ATn = average exposure time for non-carcinogens (EF × ED) (365 days/year for 30 years, ATn = 10,950 days)
RfD = oral reference dose of the potentially toxic element (mg/kg/day) (Supplementary Table S2)

The RfD is used to estimate the daily exposure level for the human population that is likely to be without appreciable risk of harmful effects over a lifetime of exposure [80], [13]. If the THQ value is below 1, the exposed human is unlikely to experience any adverse health effects. However, if the THQ value is 1 or above, there is a potential human health risk [34], [2], and necessary protective measures should be implemented. It has been reported that exposure to two or more toxic potentially toxic elements may result in additive and/or interactive effects [2].

#### 2.3.3 Hazard index (HI)

The hazard index (HI) approach, as outlined by the USEPA [78], is used to assess the overall potential non-carcinogenic risk resulting from exposure to multiple components. The HI is calculated by summing the target hazard quotients (THQs) of individual elements [80]. When the HI value exceeds 1, there is a potential significant concern for human health risks. In this study, the HI was calculated by adding the THQ values of respective potentially toxic elements in samples using the following equation:

HI = THQ (As) + THQ (Cd) + THQ (Cr) + THQ (Hg) + THQ (Pb)

Where:

HI = hazard index
THQ (As) = target hazard quotient for arsenic in diet
THQ (Cd) = target hazard quotient for cadmium in diet
THQ (Cr) = target hazard quotient for chromium in diet
THQ (Hg) = target hazard quotient for mercury in diet
THQ (Pb) = target hazard quotient for lead in diet

#### 2.3.4 Target carcinogenic risk (TCR)

Target carcinogenic risk (TCR) is used to estimate the increased probability of an individual developing cancer over a lifetime due to exposure to a potential carcinogen. The following equation is used to estimate the target carcinogenic risk [80]:

$$\mathrm{TCR}=\frac{\mathrm{EF}\times \mathrm{ED}\times \mathrm{IR}\times \mathrm{CM}\times \mathrm{CP}S_{o}}{\mathrm{AWB}\times \;AT_{c}}{\times 10}^{-3}$$

Where:

TCR = target carcinogenic risk
IR = ingestion rate of chicken samples (g/day)
CM = metal concentration (mg/kg, wet weight) in chicken samples
CPSo = carcinogenic potency slope, oral (mg/kg body weight/day)
ED = exposure duration (years)
EF = exposure frequency (days/year)
WAB = average body weight (kg)
ATc = average time for carcinogens (365 days/year for 70 years) [80]

The standard CPSo values (mg/kg body weight/day) are 0.0085 for lead, 0.38 for cadmium, 0.5 for chromium, and 1.5 for arsenic.

TCR values were calculated for intake of these potentially toxic elements. TCR values exceeding 1×10^–4^ are considered unacceptable, values below 1×10^–6^ are considered safe, and values ranging from 1×10^–4^×10^–6^ are not regarded as hazardous for humans [47]. According to the New York State Department of Health 2007, TCR values are interpreted as follows:

TCR ≤ 10^–6^: low risk
TCR between 10^-5–5^ and and 10^–4^: moderate risk
TCR between 10^-3–3^ and and 10^–1^: high risk
TCR ≥ 10^–1^: very high risk

## 3 Results and discussion

### 3.1 Lead

#### 3.1.1 Lead (Pb) in chicken

The mean concentrations of lead (Pb) in meat, liver and kidney of native, poultry and layer chickens ranged from 0.481 to 1.067 mg/kg fw (Table 1, Table 2, Table 3). The mean concentrations of Pb in the meat, liver and kidney of native, poultry and layer chickens have been shown in Table 1, Table 2, Table 3, respectively.

**Table 1:** Mean concentrations (mg/kg, fresh weight) of Pb, Cd, Cr, As and Hg in native chicken samples collected from Santosh, Tangail. Same small letters in the columns are not significantly different (n=3).

**Types of Chicken**	**Parts**	**Pb (mg/kg)**			**Cd (mg/kg)**			**Cr (mg/kg)**			**As (mg/kg)**			**Hg (mg/kg)**		
		**Mean**	**SD**	**Post hoc test**	**Mean**	**SD**	**Post hoc test**	**Mean**	**SD**	**Post hoc test**	**Mean**	**SD**	**Post hoc test**	**Mean**	**SD**	**Post hoc test**
**Native**	**Meat**	1.049	0.017	a	0.044	0.005	b	0.307	0.104	a	0.021	0.017	a	0.009	0.005	a
	**Liver**	0.483	0.245	b	0.118	0.024	a	0.134	0.044	b	0.007	0.245	a	0.011	0.024	a
	**Kidney**	0.653	0.326	ab	0.112	0.023	a	0.268	0.025	a	0.022	0.326	a	0.019	0.023	a

**Table 2:** Mean concentrations (mg/kg, fresh weight) of Pb, Cd, Cr, As and Hg in poultry chicken samples collected from Santosh, Tangail. Same small letters in the columns are not significantly different (n=3).

**Types of Chicken**	**Parts**	**Pb (mg/kg)**			**Cd (mg/kg)**			**Cr (mg/kg)**			**As (mg/kg)**			**Hg (mg/kg)**		
		**Mean**	**SD**	**Post hoc test**	**Mean**	**SD**	**Post hoc test**	**Mean**	**SD**	**Post hoc test**	**Mean**	**SD**	**Post hoc test**	**Mean**	**SD**	**Post hoc test**
**Poultry**	**Meat**	0.816	0.124	ab	0.025	0.018	a	0.319	0.096	a	0.053	0.124	ab	0.013	0.018	a
	**Liver**	0.481	0.192	b	0.026	0.006	a	0.069	0.030	b	0.032	0.192	b	0.002	0.006	b
	**Kidney**	0.886	0.229	a	0.025	0.006	a	0.253	0.084	a	0.071	0.229	a	0.007	0.006	ab

**Table 3:** Mean concentrations (mg/kg, fresh weight) of Pb, Cd, Cr, As and Hg in layer chicken samples collected from Santosh, Tangail. Same small letters in the columns are not significantly different (n=3).

**Types of Chicken**	**Parts**	**Pb (mg/kg)**			**Cd (mg/kg)**			**Cr (mg/kg)**			**As (mg/kg)**			**Hg (mg/kg)**		
		**Mean**	**SD**	**Post hoc test**	**Mean**	**SD**	**Post hoc test**	**Mean**	**SD**	**Post hoc test**	**Mean**	**SD**	**Post hoc test**	**Mean**	**SD**	**Post hoc test**
**Layer**	**Meat**	1.067	0.271	a	0.030	0.018	b	0.158	0.041	a	0.053	0.271	a	0.010	0.018	a
	**Liver**	0.511	0.063	b	0.097	0.008	a	0.220	0.110	a	0.032	0.063	a	0.004	0.008	a
	**Kidney**	0.548	0.211	b	0.093	0.007	a	0.283	0.146	a	0.055	0.211	a	0.010	0.007	a

The mean concentrations of lead (Pb) among the native, poultry and layer chicken revealed the descending order for meat, liver and kidney of: layer > native > poultry; layer > native > poultry and poultry > native > layer, respectively (Table 1, Table 2, Table 3). The highest mean concentration of Pb was obtained in layer chicken meat (1.067 mg/kg fw) (Table 3) and the lowest mean concentration of Pb was found in poultry chicken liver (0.481 mg/kg fw) (Table 2).

In this study, the mean concentrations of Pb found in analyzed meat, liver and kidney of native, poultry and layer chickens were higher than the maximum permissible limit (MPL) (0.1 mg/kg fw) set by WHO/FAO [41], Bangladesh Food Safety Authority [14] and European Commission [20] and lower than the MPL (1.0 mg/kg fw) recommended by Australia and New-Zealand Food Authority [25] (Table 4).

**Table 4:** Maximum permissible limits (MPL) (mg/kg fw) of potentially toxic elements in chicken.

**Food items**	**Toxic elements**	**Conc. (mg/kg fw)**		**References**
Chicken organs	Pb	0.1		WHO/FAO [41]
		0.1		[14]
		0.1		[20]
		1.0		[25]
	Cd		0.05	[20]
	Cr	0.1		[25]
	As	2.0		[25]
		0.05		[20]
	Hg	0.03		[25]
		0.05		[20]

FSANZ: Food Standards Australia New Zealand, EC: European Commission, BFSA: Bangladesh Food Safety Authority, WHO: Worl Health Organization, FAO: Food and Agriculture Organization.

The elevated levels of Pb mean concentrations in native, poultry and layer chicken may be attributed to the high bio accumulative characteristics of liver, kidney and meat tissues of chickens and the results are inconsistent with the findings of other authors Hossain et al. [27]; Bortey-Sam et al. [15]; Okoye et al. [63], El Bayomi et al. [22], Uluozlu et al. [76], Khan et al. [45]; Khan et al. [46]; Imran et al. [32]; Ei-Salam et al. [21]; Al-Zuhairi et al. [9] (Table 5). The Pb concentrations were observed higher in all chicken parts probably due to the high concentration of Pb in chicken feed.

**Table 5:** Comparison of Pb (mg/kg fw) in selected parts of several chickens with the reported values in the literatures. For this study, n=3 was used.

**Parts**	**Types**	**Pb conc.**	**References**	**Region**
**Meat**	***Native***	***1.049 mg/kg fw***	***This study***	***Bangladesh***
	***Poultry***	***0.816 mg/kg fw***		
	***Layer***	***1.067 mg/kg fw***		
	Poultry	3.15 mg/kg	[56]	Pakistan
	Chicken	3.1 mg/kg fw		
	Poultry	3.1 mg/kg	[56]	
	Chicken	0.41 μg/g	[49]	India
		0.011 mg/kg ww	[15]	Ghana
	Poultry	0.032 mg/kg, 0.033 mg/kg, 0.040 mg/kg, 0.038 mg/kg, 0.037 mg/kg, 0.024 mg/kg	[28]	Bangladesh
	Chicken	0.046 mg/g	[63]	Nigeria
		0.058 mg/g		
		0.77 mg/kg	[38]	
	Poultry	0.29 g/g dw	[74]	Palestine
	Chicken	0.25 μg/gm	[23]	Egypt
		0.10 μg/kg	[22]	
**Liver**	***Native***	***0.483 mg/kg fw***	***This study***	***Bangladesh***
	***Poultry***	***0.481 mg/kg fw***		
	***Layer***	***0.511 mg/kg fw***		
	Chicken	2.29 mg/kg	[32]	Pakistan
		0.99 mg/kg	[45]	
	Poultry	3.15 mg/kg	[56]	
	Chicken	2.9 mg/kg	[21]	
	Poultry	0.082 μg/g dw	[81]	Philippines
	Chicken	0.95 μg/g	[49]	India
	Layer	0.411 mg/g	[63]	Nigeria
	Broiler	0.370 mg/g		
	Chicken	0.503 mg/g		
	Layer	0.21 μg/kg ww	[22]	Egypt
	Chicken	0.56 μg/g	[62]	Uganda
		0.12 mg/kg dw	[76]	Turkey
		0.14 mg/kg	[4]	Saudi Arabia
		0.068 mg/kg ww	[30]	China
	Poultry	0.104 μg/g dw	[31]	Kuwait
**Kidney**	***Native***	***0.653 mg/kg fw***	***This study***	***Bangladesh***
	***Poultry***	***0.886 mg/kg fw***		
	***Layer***	***0.548 mg/kg fw***		
	Chicken	2.42 mg/kg	[32]	Pakistan
		3.52 μg/g dw	[74]	Palestine
	Layer	0.458 mg/g	[65]	Nigeria
	Broiler	0.243 mg/g		
	Chicken	0.478 mg/g		
	Layer	0.343 mg/g		
	Chicken	0.043 mg/kg, 0.040 mg/kg, 0.052 mg/kg, 0.053 mg/kg, 0.037 mg/kg	[28]	Bangladesh
		0.042 mg/kg ww, 0.077 mg/kg ww	[30]	China
		1.18 μg/g	[49]	India
		2.8726 mg/kg	[9]	Iraq
		0.25 mg/kg ww	[15]	Ghana

### 3.2 Cadmium (Cd)

#### 3.2.1 Cadmium (Cd) in chickens

The mean concentrations of cadmium (Cd) in meat, liver and kidney of native, poultry and layer chickens ranged from 0.025 to 0.118 mg/kg fw (Table 1, Table 2, Table 3). The mean concentrations of cadmium (Cd) in the different types of chicken organs have been shown in Table 1, Table 2, Table 3, respectively. The mean concentrations of cadmium (Cd) among the native, poultry and layer chicken showed the similar descending order for meat, liver and kidney and the order is: native > layer > poultry (Table 1, Table 2, Table 3).

In this study, the highest mean concentration of Cd was found in the liver and kidney of native and poultry chicken (Table 1, Table 2) and the lowest Cd was observed in meat, liver and kidney of poultry chicken (Table 2).

The mean concentrations of Cd found in native chicken liver (0.118 mg/kg fw), native chicken kidney (0.112 mg/kg fw) and layer chicken liver (0.097 mg/kg fw), layer chicken kidney (0.093 mg/kg fw) was higher than the MPL (0.05 mg/kg) recommended by the European Commission [20] (Table 4). The cadmium (Cd) concentrations were within the EC permissible limit (0.05 mg/kg fw) in native chicken meat (0.044 mg/kg fw) and layer chicken meat (0.030 mg/kg fw) and poultry meat (0.025 mg/kg fw), poultry liver (0.026 mg/kg fw) and poultry kidney (0.025 mg/kg fw) (Table 1, Table 2, Table 3).

The results of our study are inconsistent with concentrations of Cd found by other authors (Table 6). Studies reported that Cd is preferentially accumulated in kidneys and livers of animals and the Cd concentrations are higher in older animals than youngers [59], [89]. In this study we can also see that the kidneys and livers of chickens accumulated more Cd than meat. The high concentrations of Cd in native chicken samples may be due to the use of phosphate fertilizers in agricultural land as the phosphate fertilizers have been reported to contain high concentrations of Cd and the Cd has been observed to be accumulated by different plants [55], [54], [58].

**Table 6:** Comparison of Cd (mg/kg fw) in selected parts of several chickens with the reported values in the literatures.

**Parts**	**Types**	**Cd conc.**	**References**	**Region**
**Meat**	***Native***	***0.044 mg/kg fw***	***This study***	***Bangladesh***
	***Poultry***	***0.025 mg/kg fw***		
	***Layer***	***0.030 mg/kg fw***		
	Chicken	0.020 mg/kg ww	[35]	Bangladesh
		0.030 mg/kg fw		
		1.15 mg/kg	[21]	Pakistan
	Poultry	0.31 mg/kg	[56]	
	Chicken	0.013 mg/kg ww	[87]	Turkey
		0.7 μg/kg ww	[50]	
		0.03 μg/gm	[23]	Egypt
	Layer	0.06 μg/kg ww	[22]	
	Broiler	0.09 μg/kg ww		
	Layer	0.027 mg/g	[63]	Nigeria
	Chicken	0.37 mg/kg	[39]	
	Poultry	0.45 μg/g dw	[74]	Palestine
	Chicken	0.00509 μg/g	[7]	Iraq
		0.23 mg/kg fw	[71]	Bangladesh
		0.018 mg/kg fw	[33]	
		0.18 μg/g	[49]	India
	Layer	0.065 mg/g	[65]	Nigeria
	Broiler	0.044 mg/g		
**Liver**	***Native***	***0.118 mg/kg fw***	***This study***	***Bangladesh***
	***Poultry***	***0.026 mg/kg fw***		
	***Layer***	***0.097 mg/kg fw***		
	Poultry	0.49 mg/kg	[56]	Pakistan
	Chicken	1.213 mg/kg	[21]	
		0.015 mg/kg	[45]	
		0.22 mg/kg ww	[15]	Ghana
	Chicken	0.057 mg/kg	[87]	Turkey
	Layer	0.06 μg/kg ww	[22]	Egypt
	Broiler	0.10 μg/kg ww		
	Poultry	0.029 μg/g dw	[81]	Philippines
	Chicken	0.649 mg/g	[63]	Nigeria
	Layer	0.608 mg/g		
	Chicken	0.05 μg/g	[23]	Egypt
		0.17 μg/g	[49]	India
		0.63 μg/g dw	[74]	Palestine
	Layer	0.480 mg/kg ww	[61]	Bangladesh
	Poultry	0.089 μg/g dw	[31]	Kuwait
	Chicken	0.015 mg/kg ww, 0.019 mg/kg ww	[30]	China
**Kidney**	***Native***	***0.112 mg/kg fw***	***This study***	***Bangladesh***
	***Poultry***	***0.025 mg/kg fw***		
	***Layer***	***0.093 mg/kg fw***		
	Chicken	0.45 μg/g dw	[74]	Palestine
	Layer	0.522 mg/g	[63]	Nigeria
	Broiler	0.243 mg/g		
	Chicken	0.578 mg/g		
	Chicken	0.39 μg/g	[49]	India
		0.72 mg/kg ww	[15]	Ghana
		0.1324 mg/kg	[9]	Iraq
		0.004 mg/kg ww, 0.004 mg/kg ww	[30]	China
	Layer	0.386 mg/g	[65]	Nigeria
	Broiler	0.324 mg/g		

### 3.3 Chromium

#### 3.3.1 Chromium (Cr) in chickens

Chromium (Cr) concentrations in meat, liver and kidney of native, poultry and layer chickens ranged from 0.069 to 0.319 mg/kg fw (Table 1, Table 2, Table 3). The mean concentrations of chromium (Cr) among the native, poultry and layer chickens was observed in descending order for meat, liver and kidney of: poultry > layer > native; layer > native > poultry and native > poultry > layer, respectively (Table 1, Table 2, Table 3). The highest mean concentration of Cr was found in poultry chicken meat (0.319 mg/kg fw) and the lowest mean concentration of Cr was found in poultry chicken liver (0.069 mg/kg fw) (Table 2).

The mean concentrations (mg/kg fw) of Cr were found in meat, liver and kidney of three types of chickens were higher than the MPL (0.1 mg/kg fw) set by Australia New Zealand Food Authority [25] (Table 4). Using tannery waste in poultry feeds can transfer potentially toxic elements to the chicken and cause accumulation in the chicken liver, kidney, muscle, and bone. Thus, foods prepared using these chicken and contaminated cereals can also be contaminated with potentially toxic elements [35]. The Cr accumulation in chicken organs were also observed at Lahore, Pakistan by Mahmud et al. [53] and at Tamil Nadu, India by Sudha [73]. However, results from this study regarding Cr mean concentrations were higher than the result obtained by Yeasmin et al. [86], Aljaff et al. [7], Iwegbue et al. [39], Swaileh et al. (2005), Ei-Salam et al. [21], Bari et al. [12], Hu et al. [30] and lower than the result obtained by Shaheen et al. [71], Islam et al. [35], Ei-Salam et al. [21], Swaileh et al. [74] (Table 7).

**Table 7:** Comparison of Cr (mg/kg fw) in selected parts of several chickens with the reported values in the literatures.

**Parts**	**Types**	**Cr conc.**	**Reference**	**Region**
**Meat**	***Native***	***0.307 mg/kg fw***	***This study***	***Bangladesh***
	***Poultry***	***0.319 mg/kg fw***		
	***Layer***	***0.158 mg/kg fw***		
	Layer	0.089 mg/g	[65]	Nigeria
	Broiler	0.035 mg/g		
	Layer	0.127 mg/g	[63]	
	Broiler	0.054 mg/g		
	Chicken	0.33 mg/kg	[39]	
		0.14 mg/kg fw	[35]	Bangladesh
		2.4 mg/kg fw		
		3.6 mg/kg fw	[33]	
		2.17 mg/kg fw	[71]	
	Poultry	0.06 mg/kg, 0.048 mg/kg, 0.112 mg /kg, 0.054 mg/kg	[86]	
	Chicken	0.05 mg/kg ww	[15]	Ghana
	Poultry	0.60 μg/g dw	Swaileh et al., 2009	Palestine
	Chicken	0.075 mg/kg	[21]	Pakistan
	Poultry	0.2 mg/kg fw	[88]	UK
	Native	0.283 mg/kg	[12]	Bangladesh
	Chicken	0.08693 μg/g	[7]	Iraq
**Liver**	***Native***	***0.134 mg/kg fw***	***This study***	***Bangladesh***
	***Poultry***	***0.069 mg/kg fw***		
	***Layer***	***0.220 mg/kg fw***		
	Broiler	1.683 mg/kg ww	[61]	Bangladesh
	Layer	0.851 mg/kg ww		
	Chicken	1.4 mg/kg	[35]	
	Chicken	0.538 mg/kg	[21]	Pakistan
	Broiler	0.045 mg/g	[63]	Nigeria
	Layer	0.083 mg/g		
	Broiler	0.06 mg/kg dw	[84]	Zambia
	Layer	0.117 mg/g	[65]	Nigeria
	Broiler	0.131 mg/g		
	Chicken	0.04 mg/kg dw	[76]	Turkey
		0.18 mg/kg	[4]	Saudi Arabia
		0.086 mg/kg ww, 0.092 mg/kg ww	[30]	China
**Kidney**	***Native***	***0.268 mg/kg fw***	***This Study***	***Bangladesh***
	***Poultry***	***0.253 mg/kg fw***		
	***Layer***	***0.283 mg/kg fw***		
	Chicken	1.14 μg/g dw	[74]	Palestine
	Layer	0.111 mg/g	[63]	Nigeria
	Broiler	0.040 mg/g		
	Chicken	0.196 mg/g		
		0.770 mg/g	[65]	
		0.24 mg/kg ww	[15]	Ghana
		0.067 mg/kg ww, 0.152 mg/kg ww	[30]	Chaina

### 3.4 Arsenic

#### 3.4.1 Arsenic (As) in chickens

The arsenic (As) mean concentrations in meat, liver and kidney of native, poultry and layer chickens ranged from 0.007 to 0.071 mg/kg fw (Table 1, Table 2, Table 3). The mean concentrations of arsenic (As) among the native, poultry and layer chickens showed the descending order for meat, liver and kidney of: poultry > layer > native; poultry > layer > native and poultry > layer > native, respectively (Table 1, Table 2, Table 3). The highest mean concentration of As was found in poultry chicken kidney (0.071 mg/kg fw) (Table 2) and the lowest mean concentration of As was found in native chicken liver (0.007 mg/kg fw) (Table 1).

In this study, the mean concentrations of As in analyzed poultry chicken meat (0.053 mg/kg fw), poultry chicken kidney (0.071 mg/kg fw) and layer chicken meat (0.053 mg/kg fw), layer chicken kidney (0.055 mg/kg fw) were higher than the MPL (0.05 mg/kg fw) recommended by European Commission [20] (Table 4). The mean concentrations of As in analyzed meat, liver and kidney of three types of chickens were lower than the MPL (2.0 mg/kg fw) set by Australia New Zealand Food Authority [25] (Table 4). The usage of some feed additives, like roxarsone (3-nitro-4-hydroxyphenyl arsonic acid) is used broadly in poultry production to regulate coccidial intestinal parasites which augment arsenic loads in chicken meat and consequently adds arsenic within the environment [82]. Arsenic (As) is accumulated in animals depending upon the type of food they consume [42]. In many parts of Bangladesh groundwater is contaminated through As and may be another source of As contamination in chicken. So, feeding chicken with arsenic-rich food and/or water contributes to the accumulation of arsenic loads in chicken.

However, results from this study regarding As concentrations were higher than the result obtained by Hu et al. [30], Ysrat et al. (1999), Islam et al. [35] and lower than the result obtained by [65], Bortey-Sam et al. [15], Okoye et al. [63], Islam et al. (2018) (Table 8).

**Table 8:** Comparison of As (mg/kg fw) in selected parts of several chickens with the reported values in the literatures.

**Parts**	**Types**	**As conc.**	**Reference**	**Region**
**Meat**	***Native***	***0.021 mg/kg fw***	***This study***	***Bangladesh***
	***Poultry***	***0.053 mg/kg fw***		
	***Layer***	***0.053 mg/kg fw***		
	Chicken	0.43 mg/kg fw	[3]	Bangladesh
		0.032 mg/kg fw	[35]	
	Chicken	0.09 mg/kg		
		2.9 mg/kg fw	[33]	
		0.43 mg/kg fw	[71]	
		0.09 mg/kg	[68]	
		44.09 mg/kg fw	[56]	Pakistan
	Layer	0.082 mg/g	[63]	Nigeria
	Broiler	0.053 mg/g		
	Chicken	0.04 mg/kg ww	[15]	Ghana
	Layer	0.086 mg/g	[65]	Nigeria
	Broiler	0.076 mg/g		
**Liver**	***Native***	***0.007 mg/kg fw***	***This study***	***Bangladesh***
	***Poultry***	***0.032 mg/kg fw***		
	***Layer***	***0.032 mg/kg fw***		
	Broiler	0.642 mg/kg ww	[61]	
	Layer	0.241 mg/kg		
	Chicken	0.77 μg/g	[23]	Egypt
	Poultry	0.003 mg/kg fw	Ysart et al., 1999	UK
	Layer	0.226 mg/g	[65]	Nigeria
	Broiler	0.189 mg/g		
	Chicken	0.263 mg/g		
	Layer	0.178 mg/g	[63]	
	Chicken	0.233 mg/g		
	Poultry	46.77 mg/kg	[56]	Pakistan
	Chicken	0.06 mg/kg dw	[76]	Turkey
		0.07 mg/kg ww	[15]	Ghana
**Kidney**	***Native***	***0.022 mg/kg fw***	***This study***	***Bangladesh***
	***Poultry***	***0.071 mg/kg fw***		
	***Layer***	***0.055 mg/kg fw***		
	Chicken	0.018 mg/kg ww, 0.035 mg/kg ww	[30]	China
	Layer	0.169 mg/g	[65]	Nigeria
	Chicken	0.171 mg/g		
	Broiler	0.144 mg/g		
	Chicken	0.14 mg/kg	[15]	Ghana
		0.140 mg/g	[63]	Nigeria
	Broiler	0.119 mg/g		
	Layer	0.188 mg/g		

### 3.5 Mercury (Hg)

#### 3.5.1 Mercury (Hg) in chickens

The mercury (Hg) levels in meat, liver, and kidney samples from native, poultry, and layer chickens ranged from 0.002 to 0.019 milligrams per kilogram of fresh weight (f.w.) (mg/kg) (Table 1, Table 2, Table 3). The mean Hg concentrations exhibited a descending order among native, poultry, and layer chickens for meat, liver, and kidney tissues. The highest mean Hg concentration was found in the kidney of native chickens (0.019 mg/kg fw) (Table 1), while the lowest mean Hg concentration was observed in the liver of poultry chickens (0.002 mg/kg fw) (Table 2). The Hg concentrations in the analyzed meat, liver, and kidney samples from native, poultry, and layer chickens were lower than the Maximum Permissible Limit (MPL) of 0.03 mg/kg set by the Australia New Zealand Food Authority (FSANZ) (Table 4). These results are consistent with findings reported by other authors (Table 9).

**Table 9:** Comparison of Hg (mg/kg fw) in selected parts of several chickens with the reported values in the literatures.

**Parts**	**Types**	**Hg conc.**	**Reference**	**Region**
**Meat**	***Native***	***0.009 mg/kg fw***	***This study***	***Bangladesh***
	***Poultry***	***0.013 mg/kg fw***		
	***Layer***	***0.010 mg/kg fw***		
	Chicken	0.015 μg/g dw, 0.009 μg/g dw	[10]	Algeria
		0.19 μg/gm	[23]	Egypt
		0.62 μg/kg ww	[22]	
	Broiler	0.055 mg/g	[65]	Nigeria
	Layer	0.040 mg/g		
	Chicken	0.071 mg/g	[63]	
	Layer	0.053 mg/g		
	Broiler	0.052 mg/g		
	Chicken	0.01 mg/kg ww	[15]	Ghana
	Chicken	0.011 g/g dw, 0.015 g/g dw, 0.015 g/g dw, 0.009 g/g dw	[8]	Saudi Arabia
**Liver**	***Native***	***0.011 mg/kg fw***	***This study***	***Bangladesh***
	***Poultry***	***0.002 mg/kg fw***		
	***Layer***	***0.004 mg/kg fw***		
	Chicken	76.01 mg/kg	[56]	Pakistan
		0.34 μg/g	[23]	Egypt
	Layer	0.41 μg/kg ww	[22]	
	Broiler	0.65 μg/kg ww		
	Broiler	0.0508 mg/g	[63]	Nigeria
	Layer	0.520 mg/g		
	Chicken	0.469 mg/g	[65]	
	Broiler	0.394 mg/g		
**Kidney**	***Native***	***0.019 mg/kg fw***	***This study***	***Bangladesh***
	***Poultry***	***0.007 mg/kg fw***		
	***Layer***	***0.010 mg/kg fw***		
	Layer	0.541 mg/g	[63]	Nigeria
	Broiler	0.343 mg/g		
	Layer	0.429 mg/g	[65]	
	Broiler	0.365 mg/g		
	Chicken	0.454 mg/g		
		0.12 mg/kg ww	[15]	Ghana

### 3.6 Potentially toxic elements in chicken feeds

#### 3.6.1 Lead (Pb) in chicken feeds

The mean concentrations of lead (Pb) in the chicken feed samples ranged from 3.731 to 5.160 mg/kg (Table 10). The feeds exhibited decreasing Pb levels in the following order: CF3 > CF2 > CF1 (Table 10). The European Union EU [24] has set a maximum permissible limit (MPL) of 5.0 mg/kg for lead in chicken feed. The mean Pb concentration in CF3 (5.160 mg/kg) exceeded this MPL. This higher level in CF3 may be attributed to the addition of tannery wastes, which are known to contain high lead concentrations, in the preparation of chicken feeds in Bangladesh [43]. In contrast, the mean Pb concentrations in CF1 (3.731 mg/kg) and CF2 (4.780 mg/kg) were below the EU's MPL. These results are inconsistent with previous findings by Bari et al. [12], Jothi et al. [43], Mottalib et al. [60], Okoye et al. [64], and Alexieva et al. [6] (Table 11).

**Table 10:** Mean concentrations (mg/kg) of potentially toxic elements in chicken feed samples collected from Santosh, Santosh, Tangail.

**Samples**	**Pb (mg/kg)**		**Cd (mg/kg)**		**Cr (mg/kg)**		**As (mg/kg)**		**Hg (mg/kg)**	
	Mean	SD	Mean	SD	Mean	SD	Mean	SD	Mean	SD
CF1	3.731	0.05	0.083	0.01	1.310	0.08	0.160	0.005	0.043	0.004
CF2	4.780	0.18	0.084	0.00	2.110	0.05	0.117	0.009	0.028	0.000
CF3	5.160	0.18	0.060	0.01	1.610	0.05	0.046	0.001	0.025	0.000
***EU, 2013***	**5**		**0.5**		**-**		**2**		**0.1**

**Table 11:** Comparison of potentially toxic elements in several chicken feeds with the reported values in the literatures.

**Sample**	**Types**	**Conc. (mg/kg)**	**Reference**	**Region**
Pb	***Chicken feed 1***	***3.731 mg/kg***	***This study***	***Bangladesh***
	***Chicken feed 2***	***4.780 mg/kg***		
	***Chicken feed 3***	***5.160 mg/kg***		
	Poultry feed	0.482 mg/kg, 0.543 mg/kg, 0.522 mg/kg, 0.580 mg/kg, 0.417 mg/kg, 0.268 mg/kg	[28]	Bangladesh
		3.78 mg/kg	[32]	
	Chicken feed	1.22 mg/kg, 0.68 mg/kg	[5]	USA
	Poultry feed	14.36 mg/kg	[60]	Bangladesh
	Chicken feed	10.32 mg/kg, 10.27 mg/kg, 10.36 mg/kg	[12]	
		0.03 mg/kg, 0.05 mg/kg		
		4.80 mg/kg	[6]	Bulgaria
	Poultry feed	4.77 mg/kg		
	Layer feed	6.99 mg/kg	[64]	Nigeria
Cd	***Chicken feed 1***	***0.083 mg/kg***	***This study***	***Bangladesh***
	***Chicken feed 2***	***0.084 mg/kg***		
	***Chicken feed 3***	***0.060 mg/kg***		
	Poultry feed	0.031 g/kg	[75]	Nigeria
	Poultry feed	0.44 mg/kg	[32]	Bangladesh
		0.45 mg/kg	[60]	
		0.41 mg/kg	[6]	Bulgaria
		0.27 mg/kg		
	Layer feed	0.435 mg/kg	[64]	Nigeria
Cr	***Chicken feed 1***	***1.310 mg/kg***	***This study***	***Bangladesh***
	***Chicken feed 2***	***2.110 mg/kg***		
	***Chicken feed 3***	***1.610 mg/kg***		
	Poultry feed	2.73 mg/kg	[6]	Bulgaria
	Broiler feed	2.26 mg/kg		
	Layer feed	1.98 mg/kg	[68]	Bangladesh
	Broiler feed	1.71 μg/g, 1.64 μg/g, 1.175 μg/g	[37]	
	Poultry feed	1.93 mg/kg	[32]	
	Poultry feed	0.529 g/kg	[75]	Nigeria
As	***Chicken feed 1***	***0.160***	***This study***	***Bangladesh***
	***Chicken feed 2***	***0.117***		
	***Chicken feed 3***	***0.046***		
	Poultry feed	0.172 mg/kg	[6]	Bulgaria
		0.170 mg/kg		
		0.303 mg/kg		
	Broiler feed	0.079 mg/kg		
Hg	***Chicken feed 1***	***0.046***	***This study***	***Bangladesh***
	***Chicken feed 2***	***0.028***		
	***Chicken feed 3***	***0.025***		
	Poultry feed	0.001 mg/kg	[6]	Bulgaria
	Chicken	16.5 g/kg, 12.2 g/kg, 8.57 g/kg	[70]	Pakistan

#### 3.6.2 Cadmium (Cd) in chicken feeds

The mean concentrations of cadmium (Cd) in the chicken feeds ranged from 0.060 to 0.084 mg/kg (Table 10). The mean concentrations (mg/kg fresh weight) of cadmium (Cd) in this study's chicken feed samples decreased in the following order: CF2 > CF1 > CF3 (Table 10). The mean concentrations of cadmium (Cd) in all chicken feeds were lower than the maximum permissible limit (MPL) of 0.5 mg/kg stipulated by the European Union EU [24] (EU, 2013). However, the results from this study regarding mean concentrations of cadmium (Cd) were higher than the result obtained by Ukpe and Chokor (2018) and lower than the results obtained by Mottalib et al. [60] and Okoye et al. [64] (Table 11). The results imply that the Cd concentrations in chicken organs probably did not increase due to the concentration of Cd in chicken feeds but probably from nearby agricultural crops that received phosphate fertilizers high in Cd concentration.

#### 3.6.3 Chromium (Cr) in chicken feeds

The chromium (Cr) mean concentrations in the chicken feeds ranged from 1.31 to 2.11 mg/kg (Table 10). The mean concentration (mg/kg) of chromium (Cr) in this study of chicken feed samples decreased in the order of: CF2 > CF3 > CF1 (Table 10). The highest mean concentration of Cr (2.11 mg/kg) was found in CF2, and the lowest mean concentration of Cr (1.31 mg/kg) was found in CF1. However, the results from this study regarding Cr concentrations were higher than the results obtained by Imran et al. (2015), Islam et al. [37], and Rashid et al. [68], but lower than the result obtained by Alexieva et al. [6] (Table 11).

#### 3.6.4 Arsenic in chicken feeds

Arsenic (As) mean concentrations in the chicken feeds ranged from 0.046 to 0.160 mg/kg (Table 10). The mean concentrations of arsenic (As) in the three chicken feeds were found lower than the maximum permissible limit (2.0 mg/kg) set by the European Union EU [24] (EU, 2013) (Table 10) and consistent with other authors (Table 11).

#### 3.6.5 Mercury (Hg) in chicken feeds

Mercury (Hg) concentrations in the chicken feeds ranged from 0.025 to 0.043 mg/kg (Table 10). The mean concentrations of Hg in CF1, CF2, and CF3 were found to be 0.043 mg/kg, 0.028 mg/kg, and 0.028 mg/kg, respectively (Table 10). Detailed mean concentrations of mercury (Hg) in various chicken feeds are presented in Table 10.

The mean concentration of mercury (Hg) in all chicken feeds in this study was lower than the maximum permissible limit (MPL) of 0.1 mg/kg recommended by the European Union (EU) in 2013 (Table 10). However, the Hg concentrations observed in this study were higher than those reported by Shah et al. [70] and Alexieva et al. [6] (Table 11).

## 4 Health risk estimation (Non-carcinogenic and carcinogenic risk)

### 4.1 Health risk assessment process

Health risk assessment is a systematic process to evaluate the potential health effects from exposure to hazardous substances. In this case, it measures the prospective health risks associated with consuming various chicken organs that may contain potentially toxic elements. The assessment employs two key metrics: Target Hazard Quotients (THQs) and the Hazard Index (HI). These are used to quantify the non-carcinogenic risks arising from the consumption of different chicken organs, such as meat, liver, and kidney. It is important to note that the THQ estimation process does not provide a quantitative estimation of the probability that an exposed population will experience adverse health effects. Instead, it serves as an indicator of the level of risk posed by the contaminant exposure. While the THQ calculation cannot precisely quantify the likelihood of adverse health outcomes, it offers valuable insights into the relative level of risk associated with consuming contaminated chicken organs. This information can guide risk management strategies and inform decisions aimed at minimizing potential health hazards.

#### 4.1.1 Target hazard quotients (THQs)

The risk associated with the carcinogenic effects of a potentially toxic element is evaluated as the likelihood of contracting cancer over a lifetime of seventy years. The Target Hazard Quotient (THQ) values for the respective potentially toxic elements through consumption of various chicken organs are presented in Table 12, Table 13, Table 14. When the THQ value is lower than 1, the risk of non-carcinogenic toxic effects is considered to be minimal (USEPA, 2018). If the THQ is equal to or greater than 1, there is a potential significant human health risk, necessitating defensive measures and proactive interventions [34], [2], [83].

**Table 12:** Target hazard quotient (THQ) for different potentially toxic elements and their hazard index (HI) from the consumption of several types of chicken meats.

**Toxic elements**	**Rfd (mg/kg)**	**Target hazard quotient (THQ)**			**Hazard index (HI)**		
		**Native Meat**	**Poultry Meat**	**Layer** **Meat**	**Native Meat**	**Poultry Meat**	**Layer** **Meat**
Pb	0.004	0.185	0.144	0.189	0.343	0.368	0.377
Cd	0.001	0.031	0.017	0.022			
Cr	0.003	0.072	0.075	0.037			
As	0.0003	0.051	0.126	0.125			
Hg	0.0016	0.004	0.006	0.004

**Table 13:** Target hazard quotient (THQ) for different potentially toxic elements and their hazard index (HI) from the consumption of several types of chicken livers.

**Toxic elements**	**Rfd (mg/kg)**	**Target hazard quotient (THQ)**			**Hazard index (HI)**		
		**Native Liver**	**Poultry Liver**	**Layer Liver**	**Native Liver**	**Poultry Liver**	**Layer Liver**
Pb	0.004	0.085	0.085	0.090	0.223	0.196	0.288
Cd	0.001	0.083	0.019	0.069			
Cr	0.003	0.032	0.016	0.052			
As	0.0003	0.017	0.075	0.075			
Hg	0.0016	0.005	0.001	0.002

**Table 14:** Target hazard quotient (THQ) for different potentially toxic elements and their hazard index (HI) from the consumption of several types of chicken kidneys.

**Toxic elements**	**Rfd (mg/kg)**	**Target hazard quotient (THQ)**			**Hazard index (HI)**		
		**Native Kidney**	**Poultry Kidney**	**Layer Kidney**	**Native Kidney**	**Poultry Kidney**	**Layer Kidney**
Pb	0.004	0.116	0.157	0.097	0.317	0.406	0.364
Cd	0.001	0.080	0.018	0.066			
Cr	0.003	0.063	0.060	0.067			
As	0.0003	0.051	0.168	0.130			
Hg	0.0016	0.009	0.003	0.004			

The THQ values for the examined native, poultry, and layer chicken samples were below 1 for all respective potentially toxic elements. The results indicate that ingestion of these elements through consumption of native, poultry, and layer chickens poses no significant non-carcinogenic health risk. In this study, the highest THQ values were found to be 0.189 for Pb in layer chicken meat, 0.083 for Cd in native chicken liver, 0.075 for Cr in poultry chicken meat, 0.168 for As in poultry chicken kidney, and 0.009 for Hg in native chicken kidney (Table 12, Table 13, Table 14). As the THQ values were lower than 1 (THQ < 1), this indicates that additional dietary intake over a long period might cause non-carcinogenic effects.

#### 4.1.2 Hazard index (HI)

The hazard index (HI) value reveals the cumulative non-carcinogenic effects of various elements resulting from the consumption of different chicken organs (meat, liver, and kidney). The HI values for the consumption of chicken meats, liver, and kidney from native, poultry, and layer chickens were 0.343, 0.368, 0.377; 0.223, 0.196, 0.288; and 0.317, 0.406, 0.364, respectively (Table 12, Table 13, Table 14). These HI values indicate that consumers of the analyzed chicken organs do not face hazardous health consequences, as all the HI values are below the permissible limit (HI < 1).

The specific contributions of the examined potentially toxic elements to the HI values for meat, liver, and kidney of native, poultry, and layer chickens were in the ascending order: Hg < Cd < Cr < As < Pb; Hg < As < Cr < Cd < Pb; and Hg < As < Cr < Cd < Pb, respectively. In this study, the HI values were found to be below the acceptable limit (HI < 1) for all native, poultry, and layer chicken organs. Therefore, this study suggests that long-term exposure to Pb, Cd, Cr, As, and Hg from the consumption of chicken organs might pose significant non-carcinogenic health risks for humans.

#### 4.1.3 Target carcinogenic risks (TCRs)

The target carcinogenic risks (TCRs) evolved from the ingestion of Pb, Cd, Cr, and As were enumerated since these potentially toxic elements possibly enhance both carcinogenic and non-carcinogenic effects which rely on the exposure dose. The TCR values of Pb, Cd, Cr, and As for humans due to exposure to these potentially toxic elements from consumption of different types of chicken organs (meat, liver and kidney) are presented in Table 15, Table 16 and Table 17. Target carcinogenic risk (TCR) values surpassing 1×10^−4^ are considered as intolerable, when risk value lower than 1×10^−6^ is considered as secure, and risk values range from 1×10^−4^ to 1×10^−6^ are not regarded as hazardous for human [47]. According to the New York State Department of Health 2007, the TCR values are narrated as if TCR ≤ 10^−6^ = less, TCR values range from 10^−5^ to 10^−4^ = considered as medium and values range from 10^−3^ to 10^−1^ = severe and TCR ≥ 10^−1^ = very severe. The TCR values from exposure of Pb, Cd, Cr, and As were found in the range of 1.2×10^−6^ to 2.7×10^−6^, 1.1×10^−5^ to 5.1×10^−6^, 1.0×10^−5^ to 4.8×10^−5^ and 1.5×10^−5^ to 9.8×10^−6^, respectively through the consumption of native, poultry and layer chicken organs (meat, liver and kidney). The results of the present study revealed that the TCR values of Pd, Cd and As in the analyzed chicken samples were within the adoptable range (1×10^−4^ to 1×10^−6^), indicating no cancer or hazardous health risk from the diet of native, poultry and layer chicken organs commonly consumed in Bangladesh. On the contrary, TCR values for Cr was slightly higher than the permissible range (TCR ≤ 10^−6^) indicating the medium risk of cancer due to exposure to Cr through the consumption of chicken organs (meat, liver and kidney), which was of concern. This disclose that additional diet over a long time period might cause carcinogenic consequences as the target carcinogenic risk (TCR) values were higher than the permissible range (TCR ≤ 10^−6^) [79].

**Table 15:** Target carcinogenic risk (TCR) of potentially toxic elements from the consumption of different types of chicken meats.

**Toxic elements**	**CPSo (mg/kg/day**	**Target carcinogenic risk (TCR)**		
		**Native meat**	**Poultry meat**	**Layer meat**
Pb	0.0085	2.7×10^−6^	2.1×10^−6^	2.7×10^−6^
Cd	0.38	5.1 × 10^−6^	2.8×10^−6^	3.5×10^−6^
Cr	0.5	4.7×10^−5^	4.8×10^−5^	2.4×10^−5^
As	1.5	9.8×10^−6^	2.4×10^−5^	2.4×10^−5^

**Table 16:** Target carcinogenic risk (TCR) of potentially toxic elements from the consumption of different types of chicken livers.

**Toxic elements**	**CPSo (mg/kg/day**	**Target carcinogenic risk (TCR)**		
		**Native Liver**	**Poultry Liver**	**Layer Liver**
Pb	0.0085	1.2×10^−6^	1.2×10^−6^	1.3×10^−6^
Cd	0.38	1.4×10^−5^	3.0×10^−6^	1.1×10^−5^
Cr	0.5	2.0×10^−5^	1.0×10^−5^	3.3×10^−5^
As	1.5	3.3×10^−6^	1.5×10^−5^	1.5×10^−5^

**Table 17:** Target carcinogenic risk (TCR) of potentially toxic elements from the consumption of different types of chicken kidneys.

**Toxic elements**	**CPSo (mg/kg/day**	**Target carcinogenic risk (TCR)**		
		**Native kidney**	**Poultry kidney**	**Layer kidney**
Pb	0.0085	1.7×10^−6^	2.3×10^−6^	1.4×10^−6^
Cd	0.38	1.3×10^−5^	2.9×10^−6^	1.1×10^−5^
Cr	0.5	4.1×10^−5^	3.8×10^−5^	4.3×10^−5^
As	1.5	9.8×10^−6^	3.2×10^−5^	2.5×10^−5^

#### 4.1.4 Estimated daily intakes (EDIs) of potentially toxic elements

The present study used to estimate the daily intake of potentially toxic elements (Pb, Cd, Cr, As and Hg) through consumption of meat, liver and kidney of native, poultry and layer chickens exposure to the potentially toxic elements. The findings are then compared to the respective maximum tolerable daily intake (MTDI) of examined potentially toxic elements.

The EDI of potentially toxic elements for humans through consumption of meat, liver and kidney of native, poultry and layer chickens and maximum tolerable daily intake (MTDI) are displayed in Table 18. The result revealed that the lowest daily intake of potentially toxic elements was found in mercury (Hg) and the highest daily intake of potentially toxic elements was found in lead (Pb). The total daily intake from the consumption of meat, liver and kidney of native, poultry and layer chickens showed the ascending order of Hg <As < Cd <Cr <Pb, Hg < Cd < As < Cr < Pb and Hg < As < Cd < Cr < Pb, respectively. The daily intake of potentially toxic elements through consumption of analyzed meat, liver and kidney of native, poultry and layer chicken were found below the recommended values set by RDA [69] and JECFA [40] (Table 18). This result indicated that the peoples have no significant prospective health risk associated with the intake of examined potentially toxic elements through the consumption of native, poultry and layer chickens in Bangladesh.

**Table 18:** Food ingestion rates and estimated daily intakes of potentially toxic elements (Pb, Cd, Cr, As and Hg) from consumption of meat, liver and kidney of different type of chicken consumed by Bangladeshi populations.

**Chickens parts**		**Estimated daily intakes (EDIs) (mg/kg/BW/day)**				
		**Pb**	**Cd**	**Cr**	**As**	**Hg**
**Native**	Meat	0.00074	0.00003	0.00022	0.00002	0.00001
	Liver	0.00034	0.00008	0.00010	0.00001	0.00001
	Kidney	0.00046	0.00008	0.00019	0.00002	0.00001
**Total daily intake**		**0.00154**	**0.00019**	**0.00051**	**0.00005**	**0.00003**
**Poultry**	Meat	0.00058	0.00002	0.00023	0.00004	0.00001
	Liver	0.00034	0.00002	0.00005	0.00002	0.00000
	Kidney	0.00063	0.00002	0.00018	0.00002	0.00001
**Total daily intake**		**0.00155**	**0.00006**	**0.00046**	**0.00008**	**0.00002**
**Layer**	Meat	0.00075	0.00002	0.00011	0.00004	0.00001
	Liver	0.00036	0.00007	0.00016	0.00002	0.00000
	Kidney	0.00039	0.00007	0.00020	0.00004	0.00001
**Total daily intake**		**0.0015**	**0.00016**	**0.00047**	**0.0001**	**0.00002**
**Maximum tolerable daily intake (MTDI)**		**0.21**	**0.046**	**0.2**	**0.126**	
**References**		*RDA [69]	**JECFA [40]			

* RDA (Recommended Dietary allowance)

** JECFA (Joint Experts Committee on Food Additives)

## 5 Conclusion

The mean concentrations of lead (Pb) exceeded the WHO/FAO and EC limits in the meat, liver and kidney of native, poultry and layer chickens. The mean concentrations of cadmium (Cd) exceeded the EC limits in the liver and kidney of native and layer chickens. The mean concentrations of chromium (Cr) in all samples except poultry liver were found higher than the permissible limits set by the FSANZ. The mean concentration of As in meat and kidney of poultry and layer chicken samples were found higher than the permissible limits set by the EC.

The high concentrations of Pb, Cd, Cr and As in some organs of chicken may be from the chicken feed. Estimated levels of Pb, Cd, Cr and As were higher than the maximum permissible limits which indicate these potentially toxic elements may have carcinogenic effects on the human body and can bring a potential health risk. The estimated daily intakes (EDIs) of potentially toxic elements from consumption of meat, liver, and kidney of native, poultry and layer chicken were found lower than the maximum tolerable daily intake (MTDI). The target hazard quotient (THQ), hazard index (HI) and target carcinogenic risk (TCR) values of the analyzed chicken samples were less than the acceptable limits for all individual potentially toxic elements, notifying no non-carcinogenic and cancer health risk from the diet of a single potentially toxic element through consumption of chicken. To reduce the risk of potentially toxic elements intrusion in the human body, the feeds and agricultural crop’s potentially toxic elements accumulation should be decreased. Further study is warranted to determine the potentially toxic elements accumulation in vegetables and other yard vegetables to find out the specific source of potentially toxic elements in native (free hand raised chicken).

## Ethical Approval

None

## Funding declaration

The authors did not take any specific fund to conduct the research work.

## Author statement

I, prof. Shamim Al Mamun, declare that this work has not been published anywhere. All the authors contribution has been acknowledged in the paper. I have been communicating with the Journal on behalf of all the co-authors.

## CRediT authorship contribution statement

**Shamim Al Mamun:** Writing – review & editing, Validation, Supervision, Resources, Project administration, Methodology, Investigation, Formal analysis, Data curation, Conceptualization. **Mohammad A. Islam:** Writing – original draft, Methodology, Formal analysis, Data curation, Conceptualization. **Shamshad B. Quraishi:** Writing – review & editing, Supervision, Resources. **Mohammad M. Hosen:** Writing – review & editing, Supervision, Resources. **Brett H. Robinson:** Writing – review & editing, Supervision, Methodology, Conceptualization. **Ismail M.M. Rahman:** Writing – review & editing, Supervision, Methodology.

## Declaration of Competing Interest

The authors declare the following financial interests/personal relationships which may be considered as potential competing interests: Professor Dr. Shamim Al Mamun reports administrative support was provided by Mawlana Bhashani Science and Technology University.

## Data Availability

Data will be made available on request.
