# Supplementary material for: Assessment of potentially toxic element contents in chickens and poultry feeds from Bangladesh markets: Implications for human health risk
Source: Toxicol Rep. 2024 Aug 10;13:101706. doi: 10.1016/j.toxrep.2024.101706 (PMC11375235; doi:10.1016/j.toxrep.2024.101706)
Supplement: Supplementary file 1 — Supplementary material. [file mmc1.docx]

# Supplementary Tables

**Table S1. Age and weight of the of the chicken**

| **Types of chicken** | **Age of chicken** | **Weight (total weight of chicken)** | **Mainly reared for** |
| --- | --- | --- | --- |
| Native | 11 months | 1.2- 1.5 kg | Egg/Meat |
| Poultry | 32-35 days | 1.2-1.6 kg | Meat |
| Layer | 12 months or more | 1.8- 2.1 kg | Egg |

**Table S2. RfD (Oral reference dose) of the heavy metals**

| **Metals** | **RfD (mg/kg/day)** | **Reference** |
| --- | --- | --- |
| Lead (Pb) | 0.004 | (USEPA, 2018),  (USEPA, 2010) |
| Cadmium (Cd) | 0.001 |  |
| Chromium (Cr) | 0.003 |  |
| Arsenic (As) | 0.0003 |  |
| Mercury (Hg) | 0.0016 |  |
