# Supplementary material for: Assessment of potentially toxic element contents in chickens and poultry feeds from Bangladesh markets: Implications for human health risk
Source: Toxicol Rep. 2024 Aug 10;13:101706. doi: 10.1016/j.toxrep.2024.101706 (PMC11375235; doi:10.1016/j.toxrep.2024.101706)
Supplement: Supplementary file 2 — Supplementary material. [file mmc2.docx]

**Supplementary data:**

**Table S1. Mean concentrations (mg/kg fresh weight) of Pb, Cd, Cr, As and Hg in native, poultry and layer chicken samples collected from Santosh Bazar, Santosh, Tangail, Bangladesh.**

| **Types**  **of**  **Chicken** | **Parts** | **Pb**  **(mg/kg fw)** | | **Cd**  **(mg/kg fw)** | | **Cr**  **(mg/kg fw)** | | **As**  **(mg/kg fw)** | | **Hg**  **(mg/kg fw)** | |
| --- | --- | --- | --- | --- | --- | --- | --- | --- | --- | --- | --- |
|  |  | **Mean** | **SD** | **Mean** | **SD** | **Mean** | **SD** | **Mean** | **SD** | **Mean** | **SD** |
| **Native** | **Meat** | 1.049 | 0.017 | 0.044 | 0.005 | 0.307 | 0.104 | 0.021 | 0.017 | 0.009 | 0.005 |
|  | **Liver** | 0.483 | 0.245 | 0.118 | 0.024 | 0.134 | 0.044 | 0.007 | 0.245 | 0.011 | 0.024 |
|  | **Kidney** | 0.653 | 0.326 | 0.112 | 0.023 | 0.268 | 0.025 | 0.022 | 0.326 | 0.019 | 0.023 |
| **Poultry** | **Meat** | 0.816 | 0.124 | 0.025 | 0.018 | 0.319 | 0.096 | 0.053 | 0.124 | 0.013 | 0.018 |
|  | **Liver** | 0.481 | 0.192 | 0.026 | 0.006 | 0.069 | 0.030 | 0.032 | 0.192 | 0.002 | 0.006 |
|  | **Kidney** | 0.886 | 0.229 | 0.025 | 0.006 | 0.253 | 0.084 | 0.071 | 0.229 | 0.007 | 0.006 |
| **Layer** | **Meat** | 1.067 | 0.271 | 0.030 | 0.018 | 0.158 | 0.041 | 0.053 | 0.271 | 0.010 | 0.018 |
|  | **Liver** | 0.511 | 0.063 | 0.097 | 0.008 | 0.220 | 0.110 | 0.032 | 0.063 | 0.004 | 0.008 |
|  | **Kidney** | 0.548 | 0.211 | 0.093 | 0.007 | 0.283 | 0.146 | 0.055 | 0.211 | 0.010 | 0.007 |
| ***WHO/FAO***  ***(JECF, 2005)*** | | **0.1** | | **-** | | **-** | | **-** | | **-** | |
| ***BFSA, 2017*** | | **0.1** | | **-** | | **-** | | **-** | | **-** | |
| ***ANZFA, 2008*** | | **1.0** | | **-** | | **0.1** | | **2.0** | | **0.03** | |
| ***EC, 2006*** | | **0.1** | | **0.05** | | **-** | | **0.05** | | **0.05** | |

**Table S2. Concentrations (mg/kg) of heavy metals in chicken feed samples collected from Santosh Bazar, Santosh, Tangail.**

| **Samples** | **Pb (mg/kg)** | | **Cd (mg/kg)** | | **Cr (mg/kg)** | | **As (mg/kg)** | | **Hg (mg/kg)** | |
| --- | --- | --- | --- | --- | --- | --- | --- | --- | --- | --- |
|  | Mean | SD | Mean | SD | Mean | SD | Mean | SD | Mean | SD |
| CF1 | 3.731 | 0.05 | 0.083 | 0.01 | 1.310 | 0.08 | 0.160 | 0.005 | 0.043 | 0.004 |
| CF2 | 4.780 | 0.18 | 0.084 | 0.00 | 2.110 | 0.05 | 0.117 | 0.009 | 0.028 | 0.000 |
| CF3 | 5.160 | 0.18 | 0.060 | 0.01 | 1.610 | 0.05 | 0.046 | 0.001 | 0.025 | 0.000 |
| ***EU, 2013*** | **5** | | **0.5** | | **-** | | **2** | | **0.1** | |

**Table S3. Comparison of Pb (mg/kg fw) in selected parts of several chickens with the reported values in the literatures.**

| **Parts** | **Types** | **Pb conc.** | **References** | **Region** |
| --- | --- | --- | --- | --- |
| **Meat** | ***Native*** | ***1.049 mg/kg fw*** | ***This study*** | ***Bangladesh*** |
|  | ***Poultry*** | ***0.816 mg/kg fw*** |  |  |
|  | ***Layer*** | ***1.067 mg/kg fw*** |  |  |
|  | Poultry | 3.15 mg/kg | Mariam et al., 2015 | Pakistan |
|  | Chicken | 3.1 mg/kg fw |  |  |
|  | Poultry | 3.1 ppm | Mariam et al., 2004 |  |
|  | Chicken | 0.41 μg/g | Kumar et al., 2007 | India |
|  |  | 0.011 mg/kg ww | Bortey-Sam et al., 2015 | Ghana. |
|  | Poultry | 0.032 ppm, 0.033 ppm, 0.040 ppm, 0.038 ppm, 0.037 ppm, 0.024 ppm | Hossain et al., 2014 | Bangladesh |
|  | Chicken | 0.046 mg/g | Okoye et al., 2015 | Nigeria |
|  |  | 0.058 mg/g |  |  |
|  |  | 0.77 mg/kg | Iwegbue et al., 2008 |  |
|  | Poultry | 0.29g/g dw | Swaileh et al., 2009 | Palestine |
|  | Chicken | 0.25 μg/gm | Elsharawy et al., 2015 | Egypt |
|  |  | 0.10 μg/kg | El Bayomi et al., 2018 |  |
| **Liver** | ***Native*** | ***0.483 mg/kg fw*** | ***This study*** | ***Bangladesh*** |
|  | ***Poultry*** | ***0.481 mg/kg fw*** |  |  |
|  | ***Layer*** | ***0.511mg/kg fw*** |  |  |
|  | Chicken | 2.29 mg/kg | Imran et al., 2015 | Pakistan |
|  |  | 0.99 mg/kg | Khan et al., 2015 |  |
|  | Poultry | 3.15 ppm | Mariam et al., 2004 |  |
|  | Chicken | 2.9 mg/kg | Ei-Salam et al., 2013 |  |
|  | Poultry | 0.082 μg/g dw | Villar et al., 2005 | Philippines |
|  | Chicken | 0.95 μg/g | Kumar et al., 2007 | India |
|  | Layer | 0.411 mg/g | Okoye et al., 2015 | Nigeria |
|  | Broiler | 0.370 mg/g |  |  |
|  | Chicken | 0.503 mg/g |  |  |
|  | Layer | 0.21 μg/kg ww | El Bayomi et al., 2018 | Egypt |
|  | Chicken | 0.56 μg/g | Ogwok et al., 2014 | Uganda |
|  |  | 0.12 mg/kg dw | Uluozlu et al., 2009 | Turkey |
|  |  | 0.14mg/kg | Al Bratty et al., 2018 | Saudi Arabia |
|  |  | 0.068 mg/kg ww | Hu et al., 2018 | China |
|  | Poultry | 0.104 μg/g dw | Husain et al., 1996 | Kuwait |
| **Kidney** | ***Native*** | ***0.653 mg/kg fw*** | ***This study*** | ***Bangladesh*** |
|  | ***Poultry*** | ***0.886 mg/kg fw*** |  |  |
|  | ***Layer*** | ***0.548mg/kg fw*** |  |  |
|  | Chicken | 2.42 mg/kg | Imran et al., 2015 | Pakistan |
|  |  | 3.52 μg/g dw | Swaileh et al., 2009 | Palestine. |
|  | Layer | 0.458 mg/g | Onyeka et al., 2015 | Nigeria. |
|  | Broiler | 0.243 mg/g |  |  |
|  | Chicken | 0.478 mg/g |  |  |
|  | Layer | 0.343 mg/g |  |  |
|  | Chicken | 0.043 ppm, 0.040 ppm, 0.052 ppm, 0.053 ppm,  0.037 ppm | Hossain et al., 2014 | Bangladesh |
|  |  | 0.042 mg/kg ww  0.077 mg/kg ww | Hu et al., 2018 | China |
|  |  | 1.18 μg/g | Kumar et al., 2007 | India |
|  |  | 2.8726 ppm | Al-Zuhairi et al., 2015 | Iraq |
|  |  | 0.25 mg/kg ww | Bortey-Sam et al., 2015 | Ghana |

**Table S4. Comparison of Cd (mg/kg fw) in selected parts of several chickens with the reported values in the literatures.**

| **Parts** | **Types** | **Cd conc.** | **References** | **Region** |
| --- | --- | --- | --- | --- |
| **Meat** | ***Native*** | ***0.044 mg/kg fw*** | ***This study*** | ***Bangladesh*** |
|  | ***Poultry*** | ***0.025 mg/kg fw*** |  |  |
|  | ***Layer*** | ***0.030mg/kg fw*** |  |  |
|  | Chicken | 0.020 mg/kg ww | Islam et al., 2015 | Bangladesh |
|  |  | 0.030 mg/kg fw |  |  |
|  |  | 1.15 mg/kg | Ei-Salam et al., 2013 | Pakistan |
|  | Poultry | 0.31 mg/kg | Mariam et al., 2004 |  |
|  | Chicken | 0.013 mg/kg ww | Yilmaz et al., 2012 | Turkey |
|  |  | 0.7 μg/kg ww | Kurnaz et al., 2011 |  |
|  |  | 0.03 μg/gm | Elsharawy et al., 2015 | Egypt |
|  | Layer | 0.06 μg/kg ww | El Bayomi et al., 2018 |  |
|  | Broiler | 0.09 μg/kg ww |  |  |
|  | Layer | 0.027 mg/g | Okoye et al., 2015 | Nigeria |
|  | Chicken | 0.37 mg/kg | Iwegbue et al., 2008 |  |
|  | Poultry | 0.45 μg/g dw | Swaileh et al., 2009 | Palestine |
|  | Chicken | 0.00509 μg/g | Aljaff et al., 2014 | Iraq |
|  |  | 0.23 mg/kg fw | Shaheen et al., 2016 | Bangladesh |
|  |  | 0.018 mg/kg fw | Islam et al., 2018 |  |
|  |  | 0.18 μg/g | Kumar et al., 2007 | India |
|  | Layer | 0.065 mg/g | Onyeka et al., 2015 | Nigeria |
|  | Broiler | 0.044 mg/g |  |  |
| **Liver**  **Liver** | ***Native*** | ***0.118 mg/kg fw*** | ***This study*** | ***Bangladesh*** |
|  | ***Poultry*** | ***0.026 mg/kg fw*** |  |  |
|  | ***Layer*** | ***0.097 mg/kg fw*** |  |  |
|  | Poultry | 0.49 ppm | Mariam et al., 2004 | Pakistan |
|  | Chicken | 1.213 mg/kg | Ei-Salam et al., 2013 |  |
|  |  | 0.015 mg/kg | Khan et al., 2015 |  |
|  |  | 0.22 mg/kg ww | Bortey-Sam et al., 201 | Ghana |
|  | Chicken | 0.057 mg/kg | Yilmaz et al., 2012 | Turkey |
|  | Layer | 0.06 μg/kg ww | El Bayomi et al., 2018 | Egypt |
|  | Broiler | 0.10 μg/kg ww |  |  |
|  | Poultry | 0.029 μg/g dw | Villar et al., 2005 | Philippines |
|  | Chicken | 0.649 mg/g | Okoye et al., 2015 | Nigeria |
|  | Layer | 0.608 mg/g |  |  |
|  | Chicken | 0.05 μg/g | Elsharawy et al., 2015 | Egypt |
|  |  | 0.17 μg/g | Kumar et al., 2007 | India |
|  |  | 0.63 μg/g dw | Swaileh et al., 2009 | Palestine |
|  | Layer | 0.480 mg/kg ww | Mottalib et al., 2018 | Bangladesh |
|  | Broiler | 4.25 mg/kg | Rahman et al., 2014 |  |
|  | Poultry | 0.089 μg/g dw | Husain et al., 1996 | Kuwait |
|  | Chicken | 0.015 mg/kg ww, 0.019 mg/kg ww | Hu et al., 2018 | China |
| **Kidney** | ***Native*** | ***0.112 mg/kg fw*** | ***This study*** | ***Bangladesh*** |
|  | ***Poultry*** | ***0.025 mg/kg fw*** |  |  |
|  | ***Layer*** | ***0.093 mg/kg fw*** |  |  |
|  | Chicken | 0.45 μg/g dw | Swaileh et al., 2009 | Palestine |
|  | Layer | 0.522 mg/g | Okoye et al., 2015 | Nigeria |
|  | Broiler | 0.243 mg/g |  |  |
|  | Chicken | 0.578 mg/g |  |  |
|  | Chicken | 0.39 μg/g | Kumar et al., 2007 | India |
|  |  | 0.72mg/kg ww | Bortey-Sam et al., 2015 | Ghana |
|  |  | 0.1324 ppm | Al-Zuhairi et al., 2015 | Iraq |
|  |  | 0.004 mg/kg ww, 0.004 mg/kg ww | Hu et al., 2018 | China |
|  | Layer | 0.386 mg/g | Onyeka et al., 2015 | Nigeria |
|  | Broiler | 0.324 mg/g |  |  |

**Table S5. Comparison of Cr (mg/kg fw) in selected parts of several chickens with the reported values in the literatures.**

| **Parts** | **Types** | **Cr conc.** | **Reference** | **Region** |
| --- | --- | --- | --- | --- |
| **Meat** | ***Native*** | ***0.307 mg/kg fw*** | ***This study*** | ***Bangladesh*** |
|  | ***Poultry*** | ***0.319 mg/kg fw*** |  |  |
|  | ***Layer*** | ***0.158 mg/kg fw*** |  |  |
|  | Layer | 0.089 mg/g | Onyeka et al., 2015 | Nigeria |
|  | Broiler | 0.035 mg/g |  |  |
|  | Layer | 0.127 mg/g | Okoye et al., 2015 |  |
|  | Broiler | 0.054 mg/g |  |  |
|  | Chicken | 0.33 mg/kg | Iwegbue et al., 2008 |  |
|  |  | 0.14 mg/kg fw | Islam et al., 2015 | Bangladesh |
|  |  | 2.4 mg/kg fw |  |  |
|  |  | 3.6 mg/kg fw | Islam et al., 2018 |  |
|  |  | 2.17 mg/kg fw | Shaheen et al., 2016 |  |
|  | Poultry | 0.06 mg/kg, 0.048 mg/kg, 0.112 mg /kg, 0.054 mg/kg | Yeasmin et al., 2017 |  |
|  | Chicken | 0.05 mg/kg ww | Bortey-Sam et al., 2015 | Ghana |
|  | Poultry | 0.60 μg/g dw | Swaileh et al., 2009 | Palestine |
|  | Chicken | 0.075 mg/kg | Ei-Salam et al., 2013 | Pakistan |
|  | Poultry | 0.2 mg/kg fw | Ysart et al., 1999 | UK |
|  | Native | 0.283 mg/kg | Bari et al., 2015 | Bangladesh |
|  | Chicken | 0.08693 μg/g | Aljaff et al., 2014 | Iraq |
| **Liver** | ***Native*** | ***0.134 mg/kg fw*** | ***This study*** | ***Bangladesh*** |
|  | ***Poultry*** | ***0.069 mg/kg fw*** |  |  |
|  | ***Layer*** | ***0.220 mg/kg fw*** |  |  |
|  | Broiler | 1.683 mg/kg ww | Mottalib et al., 2018 | Bangladesh |
|  | Layer | 0.851 mg/kg ww |  |  |
|  | Chicken | 1.4 mg/kg | Islam et al., 2015 |  |
|  | Chicken | 0.538 mg/kg | Ei-Salam et al., 2013 | Pakistan |
|  | Broiler | 0.045 mg/g | Okoye et al., 2015 | Nigeria |
|  | Layer | 0.083 mg/g |  |  |
|  | Broiler | 0.06 mg/kg dw | Yabe et al., 2013 | Zambia |
|  | Layer | 0.117 mg/g | Onyeka et al., 2015 | Nigeria |
|  | Broiler | 0.131 mg/g |  |  |
|  | Chicken | 0.04 mg/kg dw | Uluozlu et al., 2009 | Turkey |
|  |  | 0.18mg/kg | Al Bratty et al., 2018 | Saudi Arabia |
|  |  | 0.086 mg/kg ww, 0.092 mg/kg ww | Hu et al., 2018 | China |
| **Kidney** | ***Native*** | ***0.268 mg/kg fw*** | ***This Study*** | ***Bangladesh*** |
|  | ***Poultry*** | ***0.253 mg/kg fw*** |  |  |
|  | ***Layer*** | ***0.283 mg/kg fw*** |  |  |
|  | Chicken | 1.14 μg/g dw | Swaileh et al., 2009 | Palestine |
|  | Layer | 0.111 mg/g | Okoye et al., 2015 | Nigeria |
|  | Broiler | 0.040 mg/g |  |  |
|  | Chicken | 0.196 mg/g |  |  |
|  |  | 0.770 mg/g | Onyeka et al., 2015 |  |
|  |  | 0.24 mg/kg ww | Bortey-Sam et al., 2015 | Ghana |
|  |  | 0.067 mg/kg ww, 0.152 mg/kg ww | Hu et al., 2018 | Chaina |

**Table S6. Comparison of As (mg/kg fw) in selected parts of several chickens with the reported values in the literatures.**

| **Parts** | **Types** | **As conc.** | **Reference** | **Region** |
| --- | --- | --- | --- | --- |
| **Meat** | ***Native*** | ***0.021 mg/kg fw*** | ***This study*** | ***Bangladesh*** |
|  | ***Poultry*** | ***0.053 mg/kg fw*** |  |  |
|  | ***Layer*** | ***0.053 mg/kg fw*** |  |  |
|  | Chicken | 0.43 mg/kg fw | Ahmed et al., 2016 | Bangladesh |
|  |  | 0.032 mg/kg fw | Islam et al., 2015 |  |
|  | Chicken | 0.09 mg/kg |  |  |
|  |  | 2.9 mg/kg fw | Islam et al., 2018 |  |
|  |  | 0.43 mg/kg fw | Shaheen et al., 2016 |  |
|  |  | 0.09 ppm | Rashid et al., 2018 |  |
|  |  | 44.09 mg/kg fw | Mariam et al., 2004 | Pakistan |
|  | Layer | 0.082 mg/g | Okoye et al., 2015 | Nigeria |
|  | Broiler | 0.053 mg/g |  |  |
|  | Chicken | 0.04 mg/kg ww | Bortey-Sam et al., 2015 | Ghana |
|  | Layer | 0.086 mg/g | Onyeka et al., 2015 | Nigeria |
|  | Broiler | 0.076 mg/g |  |  |
| **Liver** | ***Native*** | ***0.007 mg/kg fw*** | ***This study*** | ***Bangladesh*** |
|  | ***Poultry*** | ***0.032 mg/kg fw*** |  |  |
|  | ***Layer*** | ***0.032 mg/kg fw*** |  |  |
|  | Broiler | 0.642 mg/kg ww | Mottalib et al., 2018 |  |
|  | Layer | 0.241 mg/kg |  |  |
|  | Chicken | 0.77 μg/g | Elsharawy et al., 2015 | Egypt |
|  | Poultry | 0.003 mg/kg fw | Ysrat et al., 1999 | UK |
|  | Layer | 0.226 mg/g | Onyeka et al., 2015 | Nigeria |
|  | Broiler | 0.189 mg/g |  |  |
|  | Chicken | 0.263 mg/g |  |  |
|  | Layer | 0.178 mg/g | Okoye et al., 2015 |  |
|  | Chicken | 0.233 mg/g |  |  |
|  | Poultry | 46.77 ppm | Mariam et al., 2004 | Pakistan |
|  | Chicken | 0.06 mg/kg dw | Uluozlu et al., 2009 | Turkey |
|  |  | 0.07 mg/kg ww | Bortey-Sam et al., 2015 | Ghana |
| **Kidney** | ***Native*** | ***0.022 mg/kg fw*** | ***This study*** | ***Bangladesh*** |
|  | ***Poultry*** | ***0.071 mg/kg fw*** |  |  |
|  | ***Layer*** | ***0.055 mg/kg fw*** |  |  |
|  | Chicken | 0.018 mg/kg ww, 0.035 mg/kg ww | Hu et al., 2018 | China |
|  | Layer | 0.169 mg/g | Onyeka et al., 2015 | Nigeria |
|  | Chicken | 0.171 mg/g |  |  |
|  | Broiler | 0.144 mg/g |  |  |
|  | Chicken | 0.14 mg/kg | Bortey-Sam et al., 2015 | Ghana |
|  |  | 0.140 mg/g | Okoye et al., 2015 | Nigeria |
|  | Broiler | 0.119 mg/g |  |  |
|  | Layer | 0.188 mg/g |  |  |

**Table S7. Comparison of Hg (mg/kg fw) in selected parts of several chickens with the reported values in the literatures.**

| **Parts** | **Types** | **Hg conc.** | **Reference** | **Region** |
| --- | --- | --- | --- | --- |
| **Meat** | ***Native*** | ***0.009 mg/kg fw*** | ***This study*** | ***Bangladesh*** |
|  | ***Poultry*** | ***0.013 mg/kg fw*** |  |  |
|  | ***Layer*** | ***0.010 mg/kg fw*** |  |  |
|  | Chicken | 0.015 μg/g dw, 0.009 μg/g dw | Badis et al., 2014 | Algeria |
|  |  | 0.19 μg/gm | Elsharawy et al., 2015 | Egypt |
|  |  | 0.62 μg/kg ww | El Bayomi et al., 2018 |  |
|  | Broiler | 0.055 mg/g | Onyeka et al., 2015 | Nigeria |
|  | Layer | 0.040 mg/g |  |  |
|  | Chicken | 0.071 mg/g | Okoye et al., 2015 |  |
|  | Layer | 0.053 mg/g |  |  |
|  | Broiler | 0.052 mg/g |  |  |
|  | Chicken | 0.01 mg/kg ww | Bortey-Sam et al., 2015 | Ghana |
|  | Chicken | 0.011 g/g dw, 0.015 g/g dw, 0.015 g/g dw, 0.009 g/g dw | Alturiqi et al., 2012 | Saudi Arabia |
| **Liver** | ***Native*** | ***0.011 mg/kg fw*** | ***This study*** | ***Bangladesh*** |
|  | ***Poultry*** | ***0.002 mg/kg fw*** |  |  |
|  | ***Layer*** | ***0.004 mg/kg fw*** |  |  |
|  | Chicken | 76.01 ppm | Mariam et al., 2004 | Pakistan |
|  |  | 0.34 μg/g | Elsharawy et al., 2015 | Egypt |
|  | Layer | 0.41 μg/kg ww | El Bayomi et al., 2018 |  |
|  | Broiler | 0.65 μg/kg ww |  |  |
|  | Broiler | 0.0508 mg/g | Okoye et al., 2015 | Nigeria |
|  | Layer | 0.520 mg/g |  |  |
|  | Chicken | 0.469 mg/g | Onyeka et al., 2015 |  |
|  | Broiler | 0.394 mg/g |  |  |
| **Kidney** | ***Native*** | ***0.019 mg/kg fw*** | ***This study*** | ***Bangladesh*** |
|  | ***Poultry*** | ***0.00 mg/kg fw*** |  |  |
|  | ***Layer*** | ***0.010 mg/kg fw*** |  |  |
|  | Layer | 0.541 mg/g | Okoye et al., 2015 | Nigeria |
|  | Broiler | 0.343 mg/g |  |  |
|  | Layer | 0.429 mg/g | Onyeka et al., 2015 |  |
|  | Broiler | 0.365 mg/g |  |  |
|  | Chicken | 0.454 mg/g |  |  |
|  |  | 0.12mg/kg ww | Bortey-Sam et al., 2015 | Ghana |

**Table S8. Comparison of heavy metals in several chicken feeds with the reported values in the literatures.**

| **Sample** | **Types** | **Conc. (mg/kg)** | **Reference** | **Region** |
| --- | --- | --- | --- | --- |
| Pb | ***Chicken feed 1*** | ***3.731 mg/kg*** | ***This study*** | ***Bangladesh*** |
|  | ***Chicken feed 2*** | ***4.780 mg/kg*** |  |  |
|  | ***Chicken feed 3*** | ***5.160 mg/kg*** |  |  |
|  | Poultry feed | 0.482 mg/kg, 0.543 mg/kg, 0.522 mg/kg, 0.580 mg/kg,  0.417 mg/kg, 0.268 mg/kg | Hossain et al., 2014 | Bangladesh |
|  | Chicken feed | 1.22 mg/kg,  0.68 mg/kg | Albu et al., 2011 | USA |
|  | Poultry feed | 14.36 mg/kg | Mottalib et al., 2016 | Bangladesh |
|  | Chicken feed | 10.32 mg/kg, 10.27 mg/kg, 10.36 mg/kg | Bari et al., 2015 |  |
|  |  | 0.03 mg/kg,  0.05 mg/kg |  |  |
|  |  | 4.80 mg/kg | Alexieva et al., 2007 | Bulgaria |
|  | Poultry feed | 4.77 mg/kg |  |  |
|  | Layer feed | 6.99 mg/kg | Okoye et al., 2011 | Nigeria |
| Cd | ***Chicken feed 1*** | ***0.083 mg/kg*** | ***This study*** | ***Bangladesh*** |
|  | ***Chicken feed 2*** | ***0.084 mg/kg*** |  |  |
|  | ***Chicken feed 3*** | ***0.060 mg/kg*** |  |  |
|  | Poultry feed | 0.031 g/kg | Ukpe et al., 2018 | Nigeria |
|  | Layer feed | 3.33 mg/kg | Rahman et al., 2014 | Bangladesh |
|  | Poultry feed |  |  |  |
|  |  | 0.45 mg/kg | Mottalib et al., 2016 |  |
|  |  | 0.41 mg/kg | Alexieva et al., 2007 | Bulgaria |
|  |  | 0.27 mg/kg |  |  |
|  | Layer feed | 0.435 mg/kg | Okoye et al., 2011 | Nigeria |
| Cr | ***Chicken feed 1*** | ***1.310 mg/kg*** | ***This study*** | ***Bangladesh*** |
|  | ***Chicken feed 2*** | ***2.110 mg/kg*** |  |  |
|  | ***Chicken feed 3*** | ***1.610 mg/kg*** |  |  |
|  | Poultry feed | 2.73 mg/kg | Alexieva et al., 2007 | Bulgaria |
|  | Broiler feed | 2.26 mg/kg |  |  |
|  | Layer feed | 1.98 ppm | Rashid et al., 2018 | Bangladesh |
|  | Broiler feed | 1.71μg/g,  1.64 μg/g,  1.175 μg/g | Islam et al., 2016 |  |
|  | Poultry feed | 0.529 g/kg | Ukpe et al., 2018 | Nigeria |
| As | ***Chicken feed 1*** | ***0.160 mg/kg*** | ***This study*** | ***Bangladesh*** |
|  | ***Chicken feed 2*** | ***0.117 mg/kg*** |  |  |
|  | ***Chicken feed 3*** | ***0.046 mg/kg*** |  |  |
|  | Poultry feed | 0.172 mg/kg | Alexieva et al., 2007 | Bulgaria |
|  |  | 0.170 mg/kg |  |  |
|  |  | 0.303 mg/kg |  |  |
|  | Broiler feed | 0.079 mg/kg |  |  |
| Hg | ***Chicken feed 1*** | ***0.046 mg/kg*** | ***This study*** | ***Bangladesh*** |
|  | ***Chicken feed 2*** | ***0.028 mg/kg*** |  |  |
|  | ***Chicken feed 3*** | ***0.025 mg/kg*** |  |  |
|  | Poultry feed | 0.001 mg/kg | Alexieva et al., 2007 | Bulgaria |
|  | Chicken | 16.5 g/kg,  12.2 g/kg,  8.57 g/kg | Shah AQ et al., 2010 | Pakistan |

**Table S9. Maximum allowable concentration (MAC) (mg/kg fw) of heavy metals in chicken.**

| **Food items** | **Heavy metal** | **Conc. (mg/kg fw)** | | **References** |
| --- | --- | --- | --- | --- |
| Chicken organs | Pb | 0.1 | | JECFA, 2005 |
|  |  | 0.1 | | EC, 2006 |
|  |  | 1.0 | | ANZFA, 2008 |
|  | Cd | | 0.05 | EC, 2006 |
|  | Cr | 0.1 | | ANZFA, 2008 |
|  | As | 2.0 | | ANZFA, 2008 |
|  |  | 0.05 | | EC, 2006 |
|  | Hg | 0.03 | | ANZFA, 2008 |

**Table S10. Target hazard quotient (THQ) for different heavy metals and their hazard index (HI) from the consumption of different types of chicken meats.**

| **Heavy**  **Metal** | **Rfd (mg/kg)** | **Target hazard quotient (THQ)** | | | **Hazard index (HI)** | | |
| --- | --- | --- | --- | --- | --- | --- | --- |
|  |  | **Native Meat** | **Poultry Meat** | **Layer**  **Meat** | **Native Meat** | **Poultry Meat** | **Layer**  **Meat** |
| Pb | 0.004 | 0.185 | 0.144 | 0.189 | 0.343 | 0.368 | 0.377 |
| Cd | 0.001 | 0.031 | 0.017 | 0.022 |  |  |  |
| Cr | 0.003 | 0.072 | 0.075 | 0.037 |  |  |  |
| As | 0.0003 | 0.051 | 0.126 | 0.125 |  |  |  |
| Hg | 0.0016 | 0.004 | 0.006 | 0.004 |  |  |  |

**Table S11. Target hazard quotient (THQ) for different heavy metals and their hazard index (HI) from the consumption of different types of chicken livers.**

| **Heavy**  **Metal** | **Rfd (mg/kg)** | **Target hazard quotient (THQ)** | | | **Hazard index (HI)** | | |
| --- | --- | --- | --- | --- | --- | --- | --- |
|  |  | **Native Liver** | **Poultry Liver** | **Layer**  **Liver** | **Native Liver** | **Poultry Liver** | **Layer**  **Liver** |
| Pb | 0.004 | 0.085 | 0.085 | 0.090 | 0.223 | 0.196 | 0.288 |
| Cd | 0.001 | 0.083 | 0.019 | 0.069 |  |  |  |
| Cr | 0.003 | 0.032 | 0.016 | 0.052 |  |  |  |
| As | 0.0003 | 0.017 | 0.075 | 0.075 |  |  |  |
| Hg | 0.0016 | 0.005 | 0.001 | 0.002 |  |  |  |

**Table S12. Target hazard quotient (THQ) for different heavy metals and their hazard index (HI) from the consumption of different types of chicken kidneys.**

| **Heavy**  **Metal** | **Rfd (mg/kg)** | **Target hazard quotient (THQ)** | | | **Hazard index (HI)** | | |
| --- | --- | --- | --- | --- | --- | --- | --- |
|  |  | **Native Kidney** | **Poultry Kidney** | **Layer**  **Kidney** | **Native Kidney** | **Poultry Kidney** | **Layer**  **Kidney** |
| Pb | 0.004 | 0.116 | 0.157 | 0.097 | 0.317 | 0.406 | 0.364 |
| Cd | 0.001 | 0.080 | 0.018 | 0.066 |  |  |  |
| Cr | 0.003 | 0.063 | 0.060 | 0.067 |  |  |  |
| As | 0.0003 | 0.051 | 0.168 | 0.130 |  |  |  |
| Hg | 0.0016 | 0.009 | 0.003 | 0.004 |  |  |  |

**Table S13. Target carcinogenic risk (TCR) of heavy metal from the consumption of different types of chicken meats.**

| **Heavy metal** | **CPSo (mg/kg/day** | **Target carcinogenic risk (TCR)** | | |
| --- | --- | --- | --- | --- |
|  |  | **Native meat** | **Poultry meat** | **Layer meat** |
| Pb | 0.0085 | 2.7×10^-6^ | 2.1×10^-6^ | 2.7×10^-6^ |
| Cd | 0.38 | 5.1 × 10^-6^ | 2.8×10^-6^ | 3.5×10^-6^ |
| Cr | 0.5 | 4.7×10^-5^ | 4.8×10^-5^ | 2.4×10^-5^ |
| As | 1.5 | 9.8×10^-6^ | 2.4×10^-5^ | 2.4×10^-5^ |

**Table S14. Target carcinogenic risk (TCR) of heavy metal from the consumption of different types of chicken livers.**

| **Heavy metal** | **CPSo (mg/kg/day** | **Target carcinogenic risk (TCR)** | | |
| --- | --- | --- | --- | --- |
|  |  | **Native Liver** | **Poultry Liver** | **Layer Liver** |
| Pb | 0.0085 | 1.2×10^-6^ | 1.2×10^-6^ | 1.3×10^-6^ |
| Cd | 0.38 | 1.4×10^-5^ | 3.0×10^-6^ | 1.1×10^-5^ |
| Cr | 0.5 | 2.0×10^-5^ | 1.0×10^-5^ | 3.3×10^-5^ |
| As | 1.5 | 3.3×10^-6^ | 1.5×10^-5^ | 1.5×10^-5^ |

**Table S15. Target carcinogenic risk (TCR) of heavy metal from the consumption of different types of chicken kidneys.**

| **Heavy metal** | **CPSo (mg/kg/day** | **Target carcinogenic risk (TCR)** | | |
| --- | --- | --- | --- | --- |
|  |  | **Native kidney** | **Poultry kidney** | **Layer kidney** |
| Pb | 0.0085 | 1.7×10^-6^ | 2.3×10^-6^ | 1.4×10^-6^ |
| Cd | 0.38 | 1.3×10^-5^ | 2.9×10^-6^ | 1.1×10^-5^ |
| Cr | 0.5 | 4.1×10^-5^ | 3.8×10^-5^ | 4.3×10^-5^ |
| As | 1.5 | 9.8×10^-6^ | 3.2×10^-5^ | 2.5×10^-5^ |

**Table S16. Food ingestion rates and estimated daily intakes of heavy metals (Pb, Cd, Cr, As and Hg) from consumption of meat, liver and kidney of different type of chicken consumed by Bangladeshi populations.**

| **Chickens parts** | | **Estimated daily intakes (EDIs) (mg/kg/BW/day)** | | | | |
| --- | --- | --- | --- | --- | --- | --- |
|  |  | **Pb** | **Cd** | **Cr** | **As** | **Hg** |
| **Native** | Meat | 0.00074 | 0.00003 | 0.00022 | 0.00002 | 0.00001 |
|  | Liver | 0.00034 | 0.00008 | 0.00010 | 0.00001 | 0.00001 |
|  | Kidney | 0.00046 | 0.00008 | 0.00019 | 0.00002 | 0.00001 |
| **Total daily intake** | | **0.00154** | **0.00019** | **0.00051** | **0.00005** | **0.00003** |
| **Poultry** | Meat | 0.00058 | 0.00002 | 0.00023 | 0.00004 | 0.00001 |
|  | Liver | 0.00034 | 0.00002 | 0.00005 | 0.00002 | 0.00000 |
|  | Kidney | 0.00063 | 0.00002 | 0.00018 | 0.00002 | 0.00001 |
| **Total daily intake** | | **0.00155** | **0.00006** | **0.00046** | **0.00008** | **0.00002** |
| **Layer** | Meat | 0.00075 | 0.00002 | 0.00011 | 0.00004 | 0.00001 |
|  | Liver | 0.00036 | 0.00007 | 0.00016 | 0.00002 | 0.00000 |
|  | Kidney | 0.00039 | 0.00007 | 0.00020 | 0.00004 | 0.00001 |
| **Total daily intake** | | **0.0015** | **0.00016** | **0.00047** | **0.0001** | **0.00002** |
| **Maximum tolerable daily intake (MTDI)** | | **0.21** | **0.046** | **0.2** | **0.126** |  |
| **References** | | ***RDA (1989)** | ****JECFA (2003)** | | |  |

***RDA (Recommended Dietary allowance)**

**** JECFA (Joint Experts Committee on Food Additives)**
